# Supplementary material for: Evidence for structural control of mare volcanism in lunar compressional tectonic settings
Source: Nat Commun. 2023 May 20;14:2892. doi: 10.1038/s41467-023-38615-1 (PMC10199890; doi:10.1038/s41467-023-38615-1)
Supplement: Supplementary file 1 — Supplementary Information [file 41467_2023_38615_MOESM1_ESM.pdf]

Supplementary Material:

Evidence for Structural Control of Mare Volcanism in Lunar Compressional Tectonic Settings

Feng Zhang[1*], Alberto Pizzi[2], Trishit Ruj[3], Goro Komatsu[2,4], An Yin[5], Yanan Dang[6], Yang Liu[1], Yongliao Zou[1]

[1]State Key Laboratory of Space Weather, National Space Science Center, Chinese Academy of Sciences, Beijing, China, [2]Department of Engineering and Geology, Università d'Annunzio, Chieti-Pescara, Italy, [3]Institute of Space and Astronautical Science (ISAS), Japan Aerospace Exploration Agency (JAXA), 3–1-1 Yoshinodai, Sagamihara, Kanagawa 252–5210, Japan, [4]International Research School of Planetary Sciences, Università d'Annunzio, Pescara, Italy, [5]Department of Earth, Planetary, and Space Sciences, University of California, Los Angeles, California 90024-1567, USA, [6]National Key Laboratory of Microwave Imaging Technology, Aerospace Information Research Institute, Chinese Academy of Sciences, Bejing, China

Correspondence and requests for materials should be addressed to [1]F. Zhang (e-mail: zhangfeng@nssc.ac.cn)

Supplementary Information

Supplementary Notes 1 to 6, Supplementary Figures 1 to 34, and Supplementary References.

Introduction

The text and figures here provide additional information on the lunar chronology, geologic units mapping, recent spacecraft data for the Moon, digitization of mare wrinkle ridges (WRs), gravity models of lunar crust thickness, and map-based geological backgrounds for the 30 candidate regions where mare volcanism-WR interactions occurred. Data used in this work are archived in the Geophysics Node of the Planetary Data System (PDS, http://pds-geosciences.wustl.edu/dataserv/moon.html) and the JAXA

KAGUYA/SELENE (http://darts.isas.jaxa.jp/planet/pdap/selene/index.html.en). The images were processed and investigated using the software ArcGIS 10.8. The same platform was employed to identify and digitize geologic features in the study regions of interest. Topography data and elevation measurements were derived from DTMs on LROC and Kaguya-TC images/sets (See Note 3 for the detail).

Note 1. Lunar Chronology

The geological time scale of the Moon is based on stratigraphic relationships of surface deposits (1) modified by large impact events, changes in crater formation through time, and impact crater size-frequency distribution measurements of specific mapped units (e.g., 2, 3). The geological time scale of the Moon is subdivided into five major periods, the Pre-Nectarian from the beginning formation of the Moon to ~3.92 Ga ago, the Nectarian ranging from ~3.92 to ~3.85 Ga ago and during this period the Nectaris Basin and other major basins were formed by large impact events, the Imbrian (Late and Early epochs) extending from ~3.85 to ~3.75 Ga (Early) and ~3.75 to ~3.2 Ga (Late), during which most maria in large impact basins formed, the Eratosthenian from ~3.2 to ~1.1 Ga ago, and the Copernican from ~1.1 Ga ago to the present. Global 1:5 M scale geological synthesis maps of the Moon can be found in Wilhelms and McCauley (4) and Fortezzo and Hare (5).

Note 2. Lunar Geological Mapping and Interpretation of Units

The age relations of lunar surface geologic units can be defined according to their superpositional and transectional relations. Geological synthesis maps of the Moon (4, 6-9) are compiled from interpretation and mapping of features and units seen in images and topography. The 1:5M near-side map published by Wilhelms and McCauley (4) was effectively a synthesis of 36 1:1M maps produced from Earth-based telescopic observations and Lunar Orbiter data sets. A general introduction to planetary data and mapping techniques can be found in Greeley and Batson (10) and Tanaka et al. (11). Descriptions of mapping techniques and major geological units for the Moon can be found in Gaddis et al. (12) and previous geological maps of the Moon referred above.

The frames of geological maps referred to in this study are listed below:

I-462 (Lambert-SE), I-385 (sinuous rilles in southern Oceanus Procellarum), I-703 (mare units, Nearside of the Moon)

Geologic Atlas of the Moon is available via the following website:

URL https://www.lpi.usra.edu/resources/mapcatalog/usgs/index.shtml

Note 3. Recent Spacecraft Data of the Moon

A host of missions to the Moon have been launched in the last decade and much of the resulting data have been utilized in this study. We used data from the following missions and instruments and the URL for each are provided below:

(1) Lunar Reconnaissance Orbiter Mission (LRO; see 13):

URL https://www.nasa.gov/mission_pages/LRO/main/index.html by NASA

URL https://lunar.gsfc.nasa.gov/ by NASA's Goddard Space Flight Center

URL https://solarsystem.nasa.gov/missions/lro/in-depth/ by NASA's Solar System Exploration

Lunar Reconnaissance Orbiter Camera (LROC): http://lroc.sese.asu.edu/

Lunar Orbiter Laser Altimeter (LOLA): https://lunar.gsfc.nasa.gov/lola/index.html

LROC WAC-derived DTMs (14): URL

https://astrogeology.usgs.gov/search/details/Moon/LRO/LROC_WAC/Lunar_LROC_WAC_GLD100_79s79n_118m_v1_1/cub

LROC WAC-derived TiO_2 abundance map (15): URL

http://wms.lroc.asu.edu/lroc/view_rdr/WAC_TIO2

(2) Gravity Recovery and Interior Laboratory mission (GRAIL; see 16):

URL https://www.nasa.gov/mission_pages/grail/main/index.html

(3) SELENE (Kaguya) mission and Terrain Camera (TC):

URL http://www.kaguya.jaxa.jp/en/equipment/tc_e.htm by PGS

(4) SELENE and LRO Elevation Model (SLDEM2015, see 17)

URL https://pds-geosciences.wustl.edu/missions/lro/lola.htm and

https://astrogeology.usgs.gov/search/map/Moon/LRO/LOLA/Lunar_LRO_LOLAKaguya_DEMmerge_60N60S_512ppd

The detailed descriptions of the data sets of these missions can be found via the following URL https://pds-imaging.jpl.nasa.gov/portal/

The LROC and Kaguya TC data (Haruyama et al., see 18) used in this work are archived in the Geophysics Node of the Planetary Data System:
URL http://pds-geosciences.wustl.edu/dataserv/moon.html.

The Kaguya TC and derived DTM data (Haruyama et al., see 19) are also available directly via the website:
URL http://darts.isas.jaxa.jp/planet/pdap/selene/

(5) The slope azimuth map derived from the SLDEM2015 data product (see 17)
Available via the online LRO Lunar Quickmap tool:
URL https://quickmap.lroc.asu.edu/

Note 4. Digitization of Lunar Mare Wrinkle Ridge

The ArcGIS software was used to digitize and display the vector map of lunar wrinkle ridges (see 20). The map base consists of a monochromatic (643 nm) LROC WAC image mosaic, optimized for geomorphology, at a resolution of 100 m/pixel. Polylines were digitized at a scale between 1:500,000 and 1:1,000,000.

The shapefile for the mare wrinkle ridges can be freely downloaded from the URL http://wms.lroc.asu.edu/lroc/view_rdr/SHAPEFILE_WRINKLE_RIDGES, and can also be accessible using the online tool QuickMap (https://quickmap.lroc.asu.edu/).

Credits: Mark Robinson, Ph.D., Principal Investigator; LROC Science Operations Center, Arizona State University; Digitized by David M. Nelson, LROC Science Operations Center, Arizona State University.

Note 5. Gravity Models of Lunar Crust Thickness

The lunar crustal thickness results of Model 1 from Wieczorek et al. (21) were

derived from GRAIL gravity data model GL0420A. The assumed crustal porosity in this model is 12%, and the mantle density is 3220 kg/m^3 (Wieczorek et al., see 21).

GRAIL Crustal Thickness Archive may be downloaded from URL https://zenodo.org/record/997347#.XEg11lMnYcM

Note 6. A General Description of Figures 6-34

Kaguya TC maps and SLDEM2015 data (see 17) are used for showing the regional geologic details of the locations where interaction between mare volcanism and wrinkle ridge-formation tectonics occurred. Some volcanic eruption centers are located on the broad topographic-rise arches of wrinkle ridges, while others in the areas linked with contractional structures (elements of WRs).

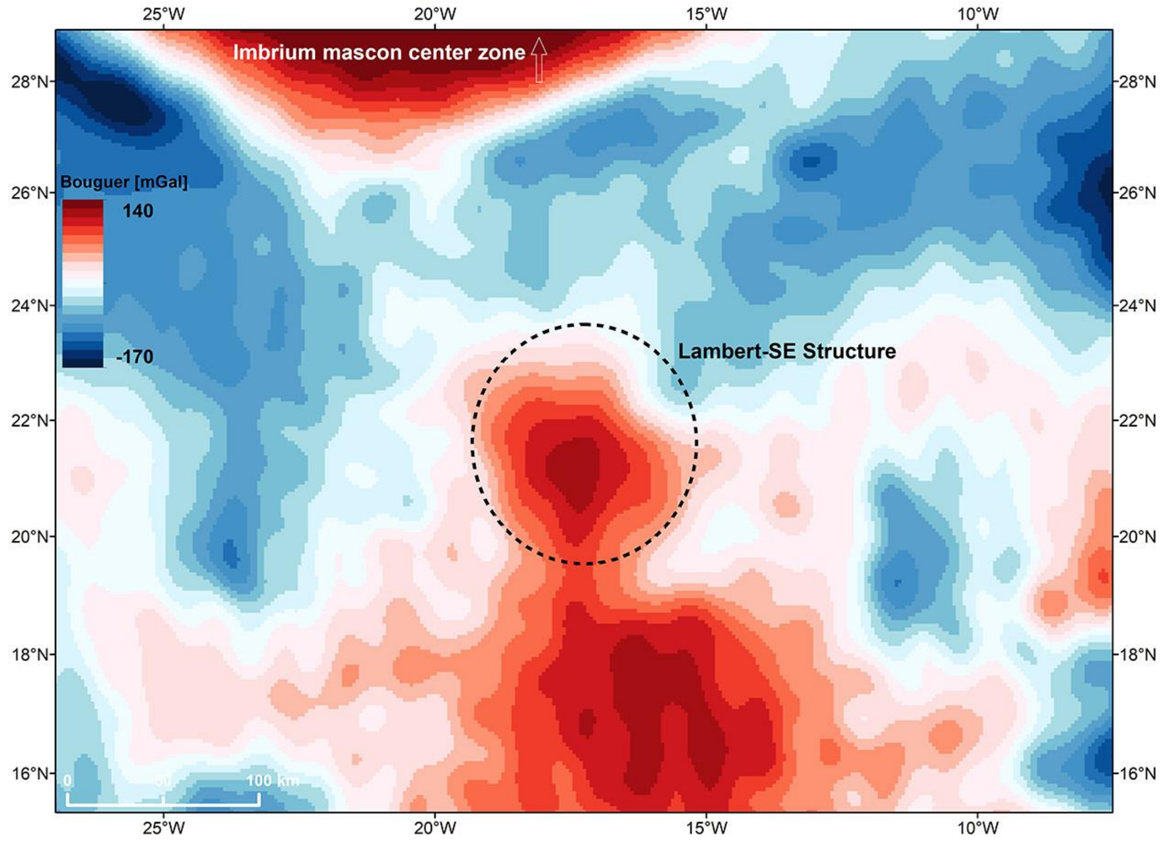

Supplementary Figure 1. Bouguer gravity anomaly map (see 22) of the area containing the Lambert-SE structure, and the dashed black line illustrates the outline of the structure.

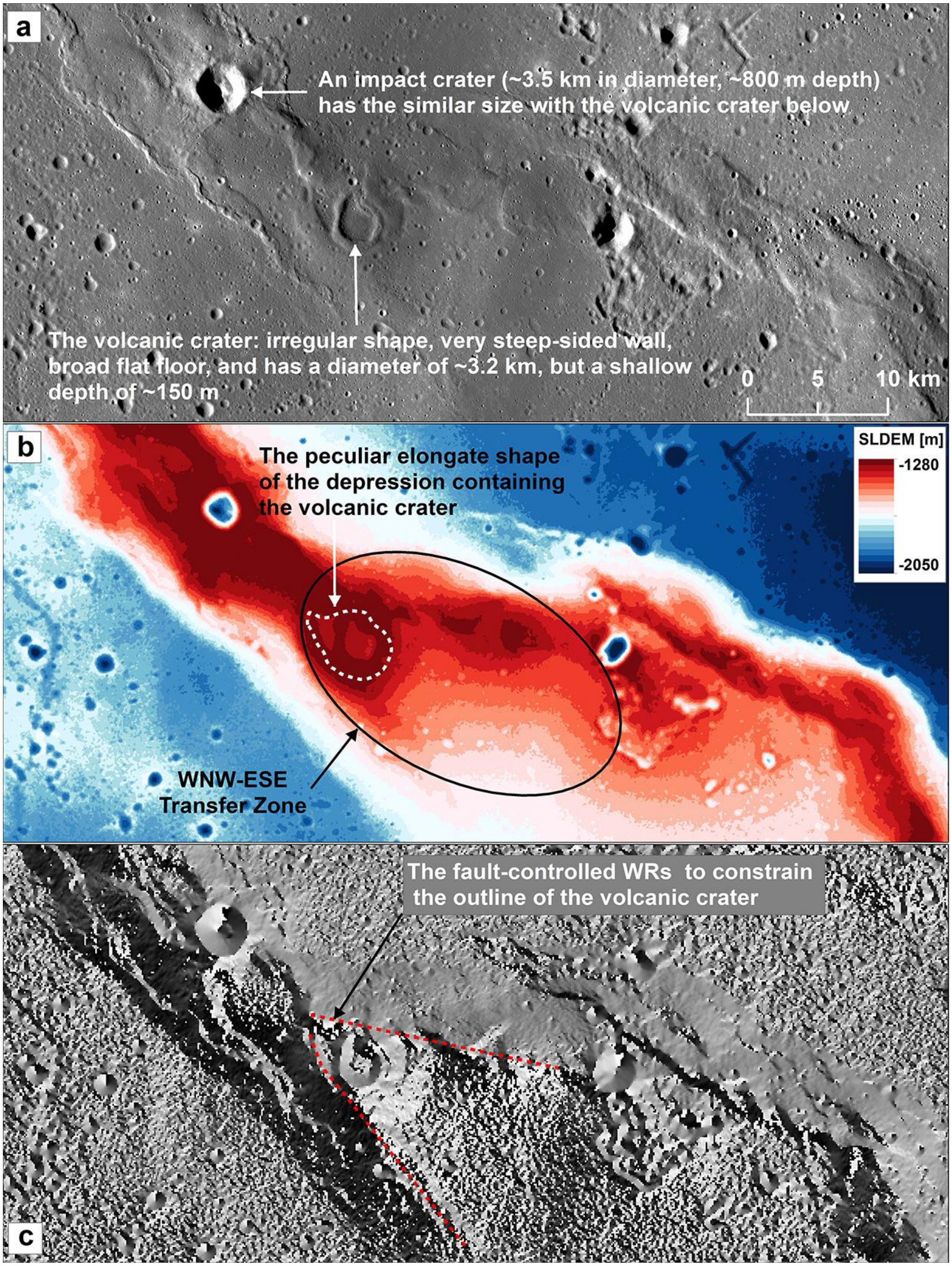

Supplementary Figure 2. (a) Kaguya TC evening map for the close-up view of the volcanic crater situated on the top of the wrinkle ridge nearly in the center of the Lambert-SE structure. It should be noted that the volcanic crater has a size similar to that of a normal impact crater to the northwest, but the volcanic crater has a flat floor with a

very shallow depth of ~150 m. (b) Colored SLDEM2015 data for the same location shown in (a). The dashed white-outline polygon illustrates the peculiar elongate shape of the depression containing the volcanic crater. The grey ellipse illustrates the transfer zone linking the two major sets of thrust faults and folds at its both sides. (c) The slope azimuth map derived from the SLDEM2015 data product (see 17) for the same region shown in (a) and (b). Note that the elongated shape of the depression within which the volcanic crater is located is well bounded by the fault-controlled WRs (dashed red lines).

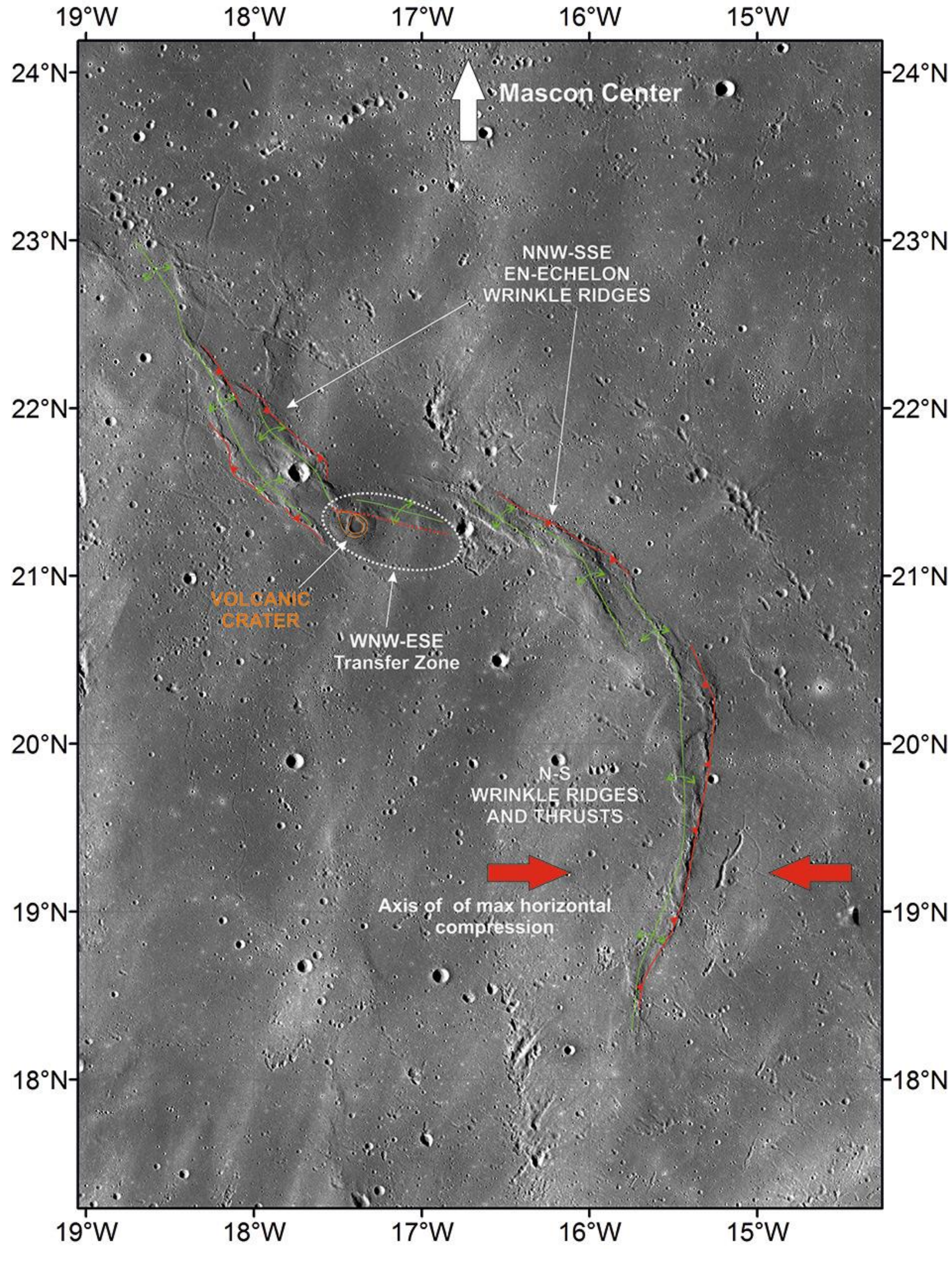

Supplementary Figure 3. Kaguya-TC evening map for the geologic background and regional stress analysis of the Lambert-SE region. Red solid lines indicate thrust faults with bar placed on the upthrown side. Green lines are delineated along crest lines of mare ridge, while green lines with arrowhead point downslope. Thick red arrows suggest axis of max horizontal contraction along E-W direction.

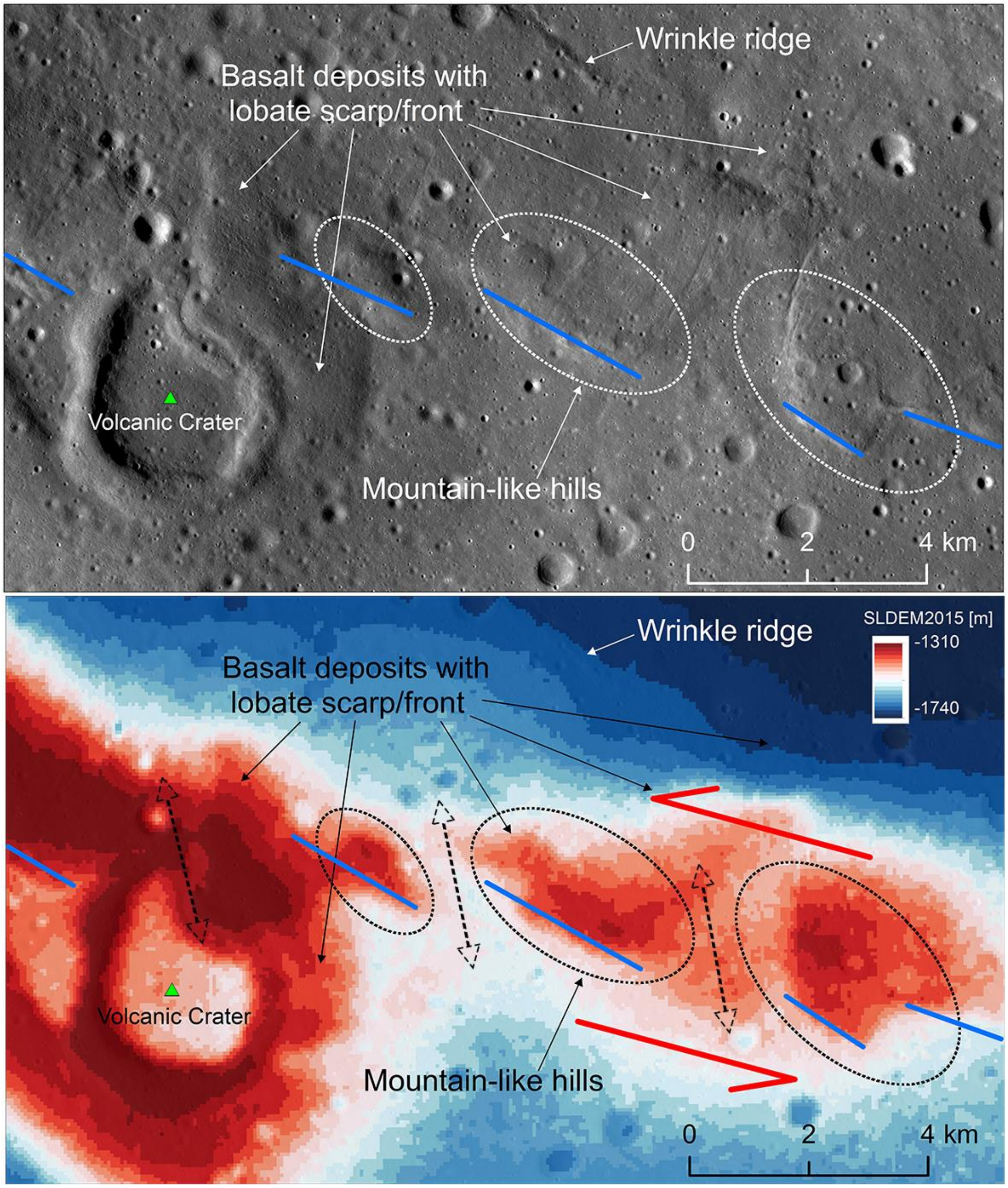

Supplementary Figure 4. The detailed geology of the transfer zone. Blue lines indicate the steep side of the NW-SE hills arranged in left stepping en echelon arrays, the envelope of which forms the WNW-ESE sinistral (red arrows) transfer zone indicated in figs. 2a, 4b and S3, coherent with the E-W max horizontal stress. Black, the dashed arrows indicate the localized extension across the step-overs, the westernmost of which is characterized by the presence of the volcanic crater.

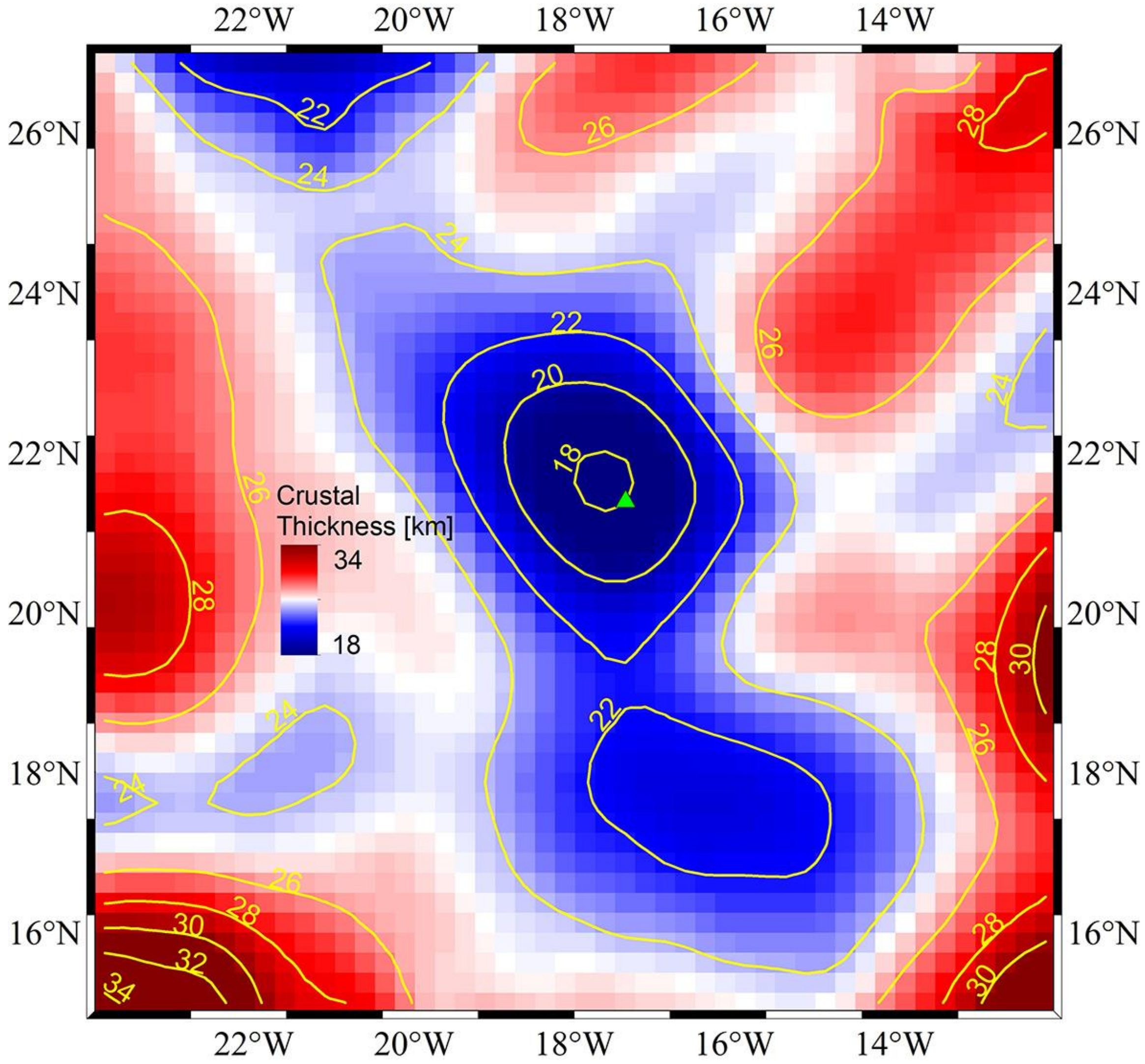

Supplementary Figure 5. Crustal thickness contours (2-km increments) are plotted for the Lambert-SE structure zone on top of GRAIL-measured crustal thickness map. Contours represent crustal thickness results of Model 1 of Wieczorek et al. (21), derived from GRAIL gravity data model GL0420A (also see Supplementary Note S5). The assumed crustal porosity in this model is 12%, and the mantle density is 3220 kg/m^3 (Wieczorek et al., see 21). Blue areas represent low crustal thickness. The green triangle indicates the volcanic crater location for local eruptions.

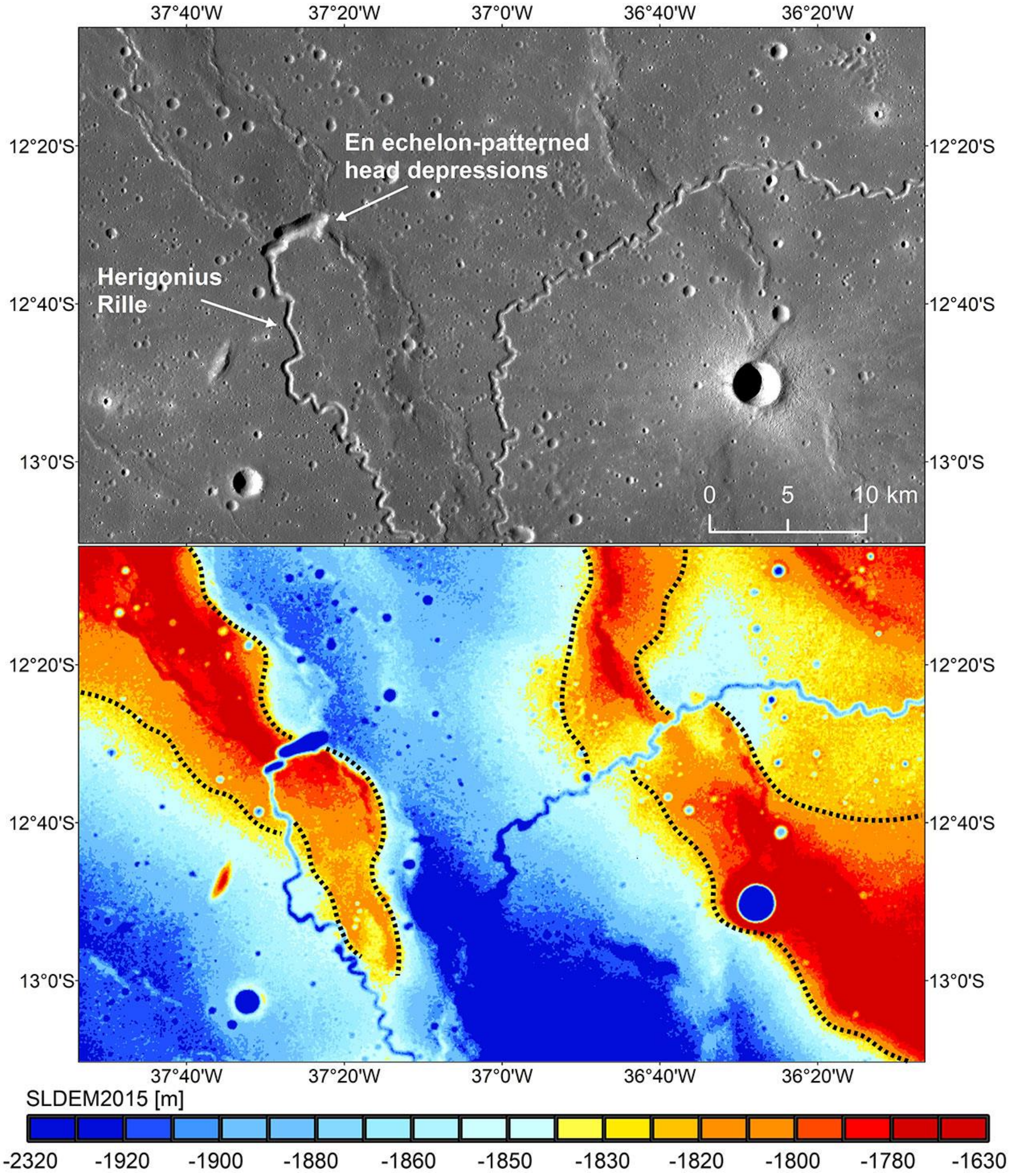

Supplementary Figure 6. Kaguya TC evening Map (top) and SLDEM2015 data (bottom) for the Herigonius rille (lava channel) in southeastern Procellarum (Location: 12.5052°S, 37.4328°W) with two elongated head depressions cutting across a wrinkle ridge (WR). (top) The two rille-head vents of elongation in shape are arranged in an en-echelon pattern and oriented along the SW-NE direction. (Bottom) SLDEM2015 data is used to show the regional topographic variation for defining the outline of broad anticlinal arches of the major WR segments. The black dashed lines illustrate the boundaries of WRs. Clearly, the two major WRs both are cut off by rilles with a certain-distance offset for their two segments, respectively. Further, this is to show the scenario for the possible sinistral strike slip movement (tear fault) along the preexisting structural weakness, e.g., basin-ring normal faults in the basement associated with the Humorum basin formation and loading tectonics.

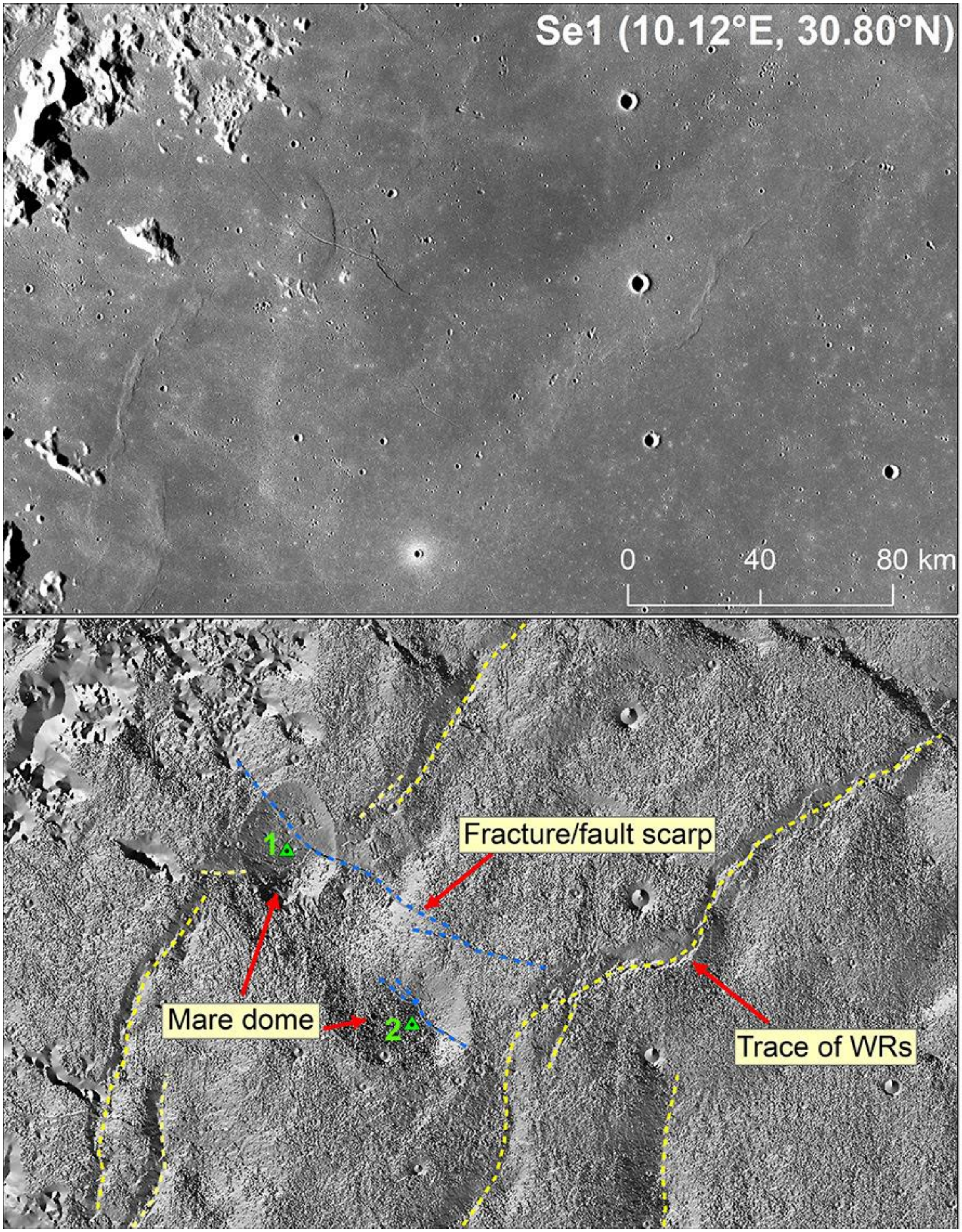

Supplementary Figure 7. The dome 1 is located at the zone where the strike of the major WR (yellow dashed line) turns to right. The dome 1 location is also an intersection place between the SW-NE-trending WR and the NW-SE fracture/normal fault (blue dashed line), indicating a subsurface tectonic control of the dome-forming eruptions.

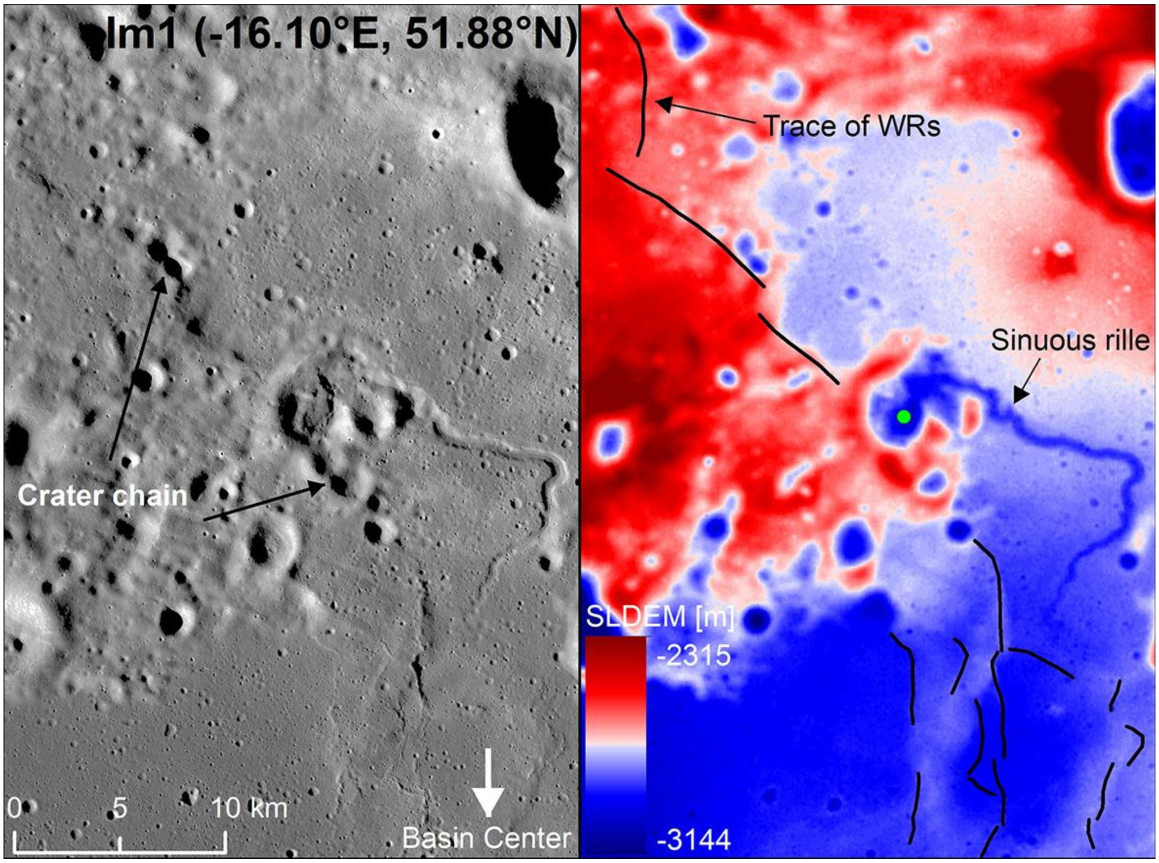

Supplementary Figure 8. Head crater (the green point) of the rille-formation flooding event is located at a possible intersection point between the WRs (black lines) and the inferred subsurface basin-concentric normal fault at the Imbrium rim/ring zone.

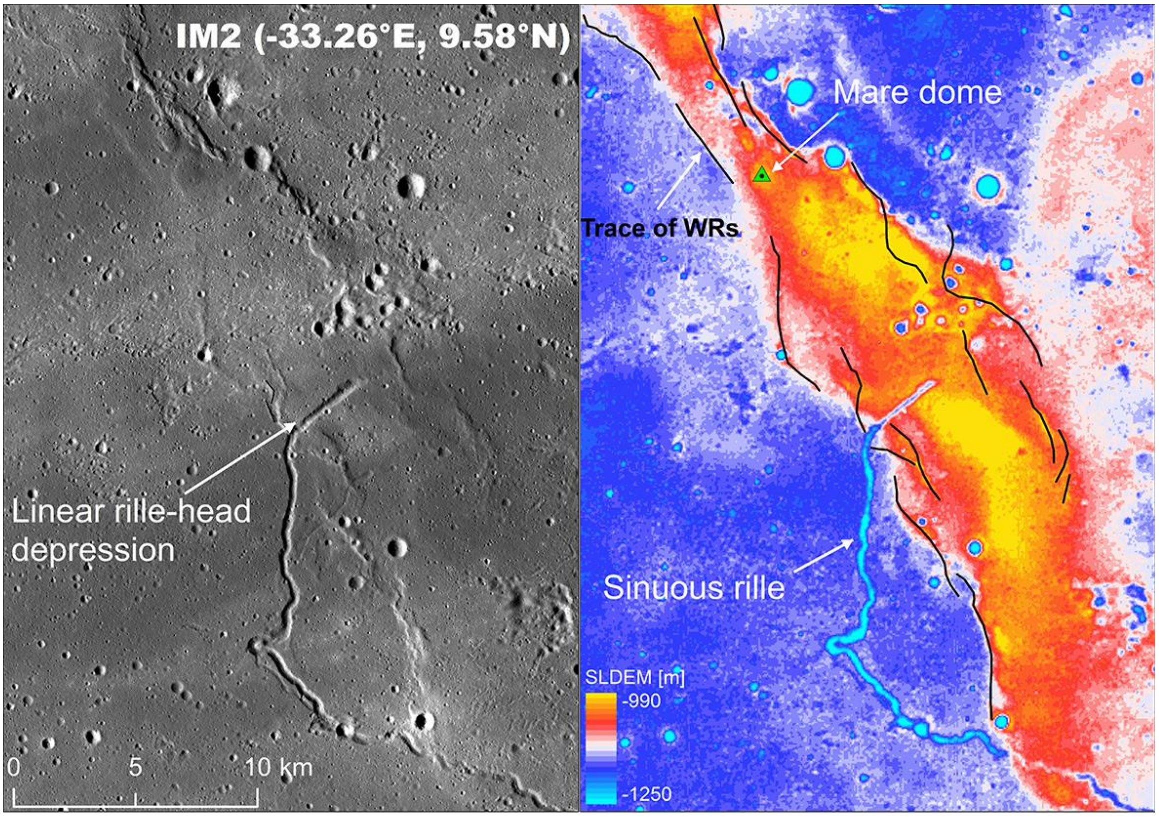

Supplementary Figure 9. The linear head depression of the rille is located on the broad arch of the ridge and radiates from the Imbrium basin, indicating a possible intersection place between the WRs and basin-radial fracture.

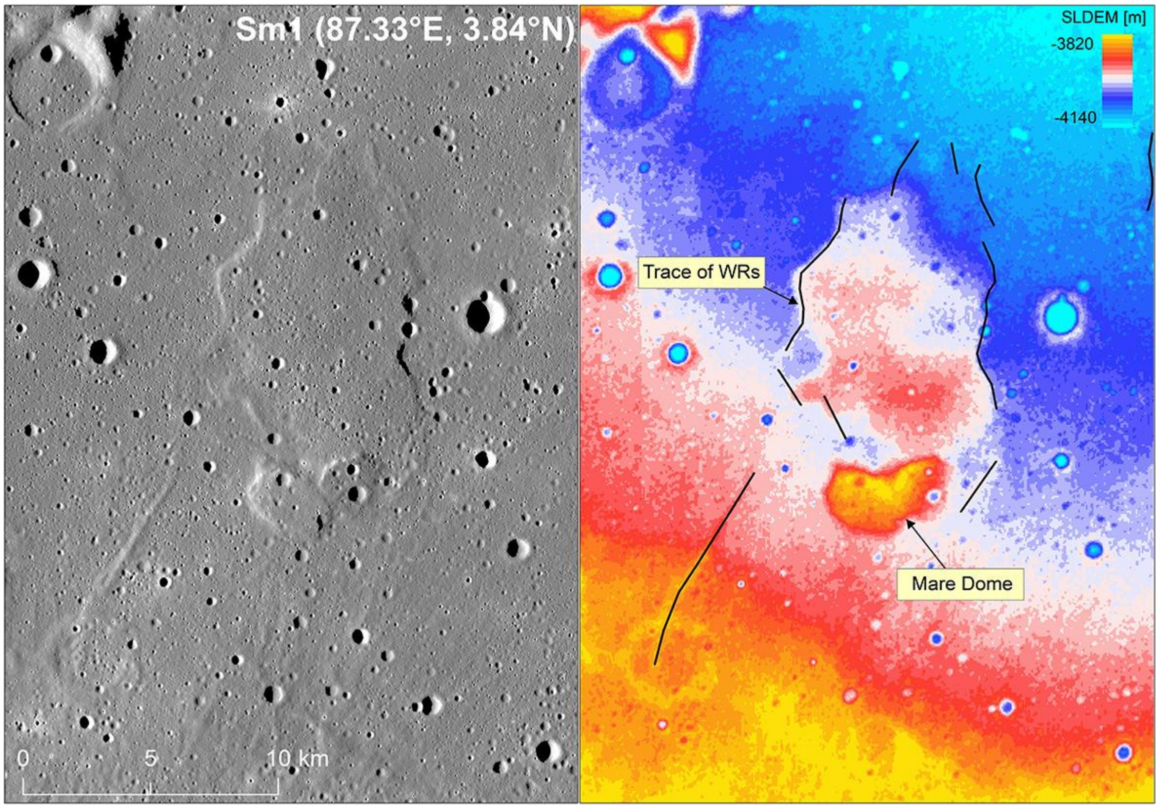

Supplementary Figure 10. The mare dome(s) occur on the broad arch of the major WR, which is radial to the Smythii basin center. The elongation of the dome(s) is along the possible subsurface basin-concentric normal faults at its rim place.

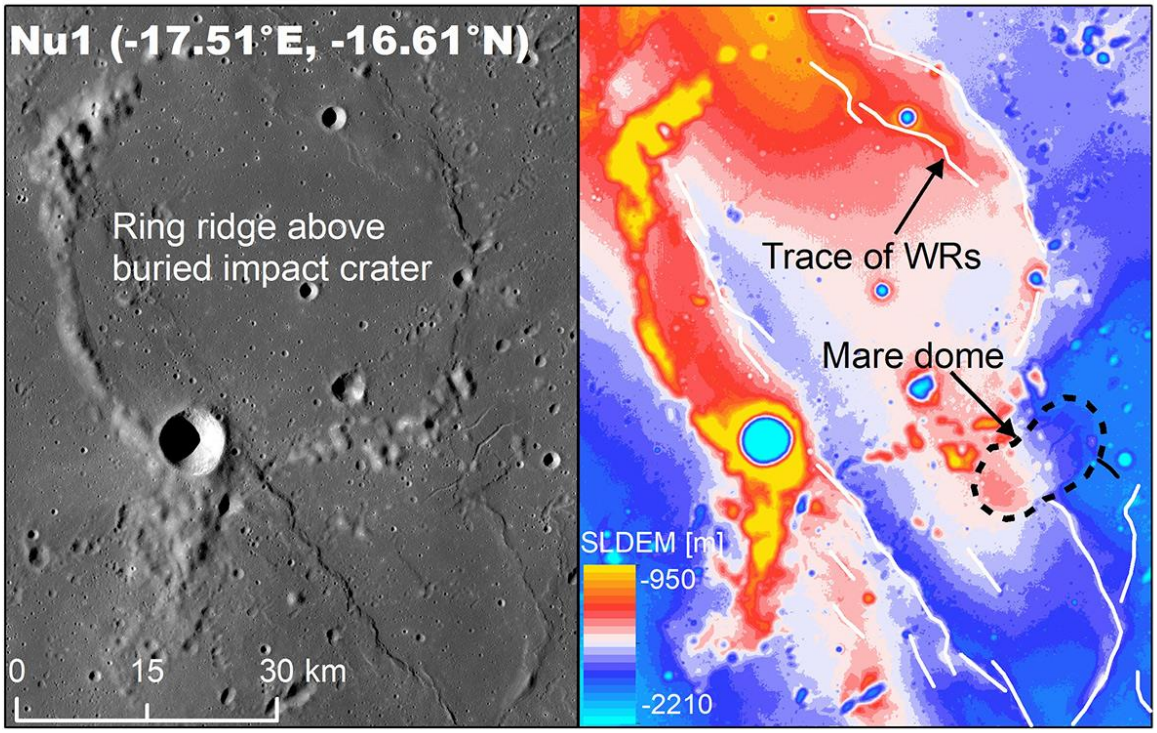

Supplementary Figure 11. The elongation of the mare dome and its top fractures (or rilles) are all concentric to the buried crater center. At the same time, its elongation is perpendicular to the WR to its southeast.

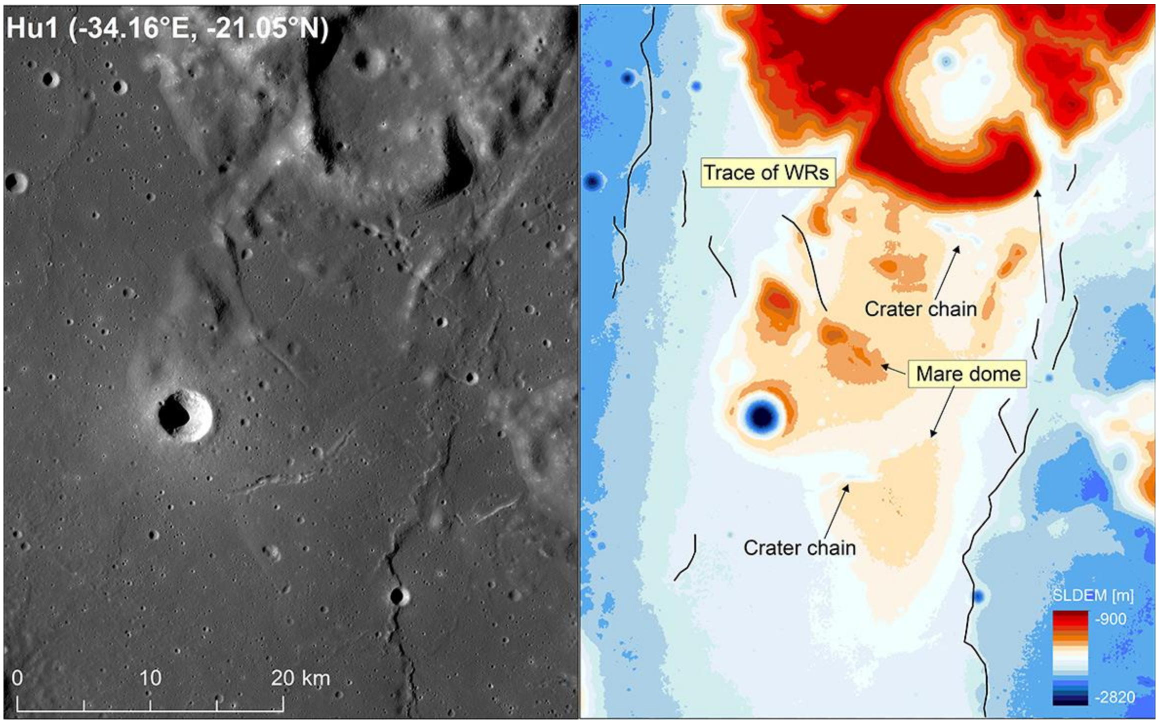

Supplementary Figure 12. The mare domes are located on the broad arch of WRs, which are concentric to the Humorum basin center. However, the elongation of the mare domes and the crater chains indicate that the eruption centers are controlled by the subsurface structures that cut across the major WR.

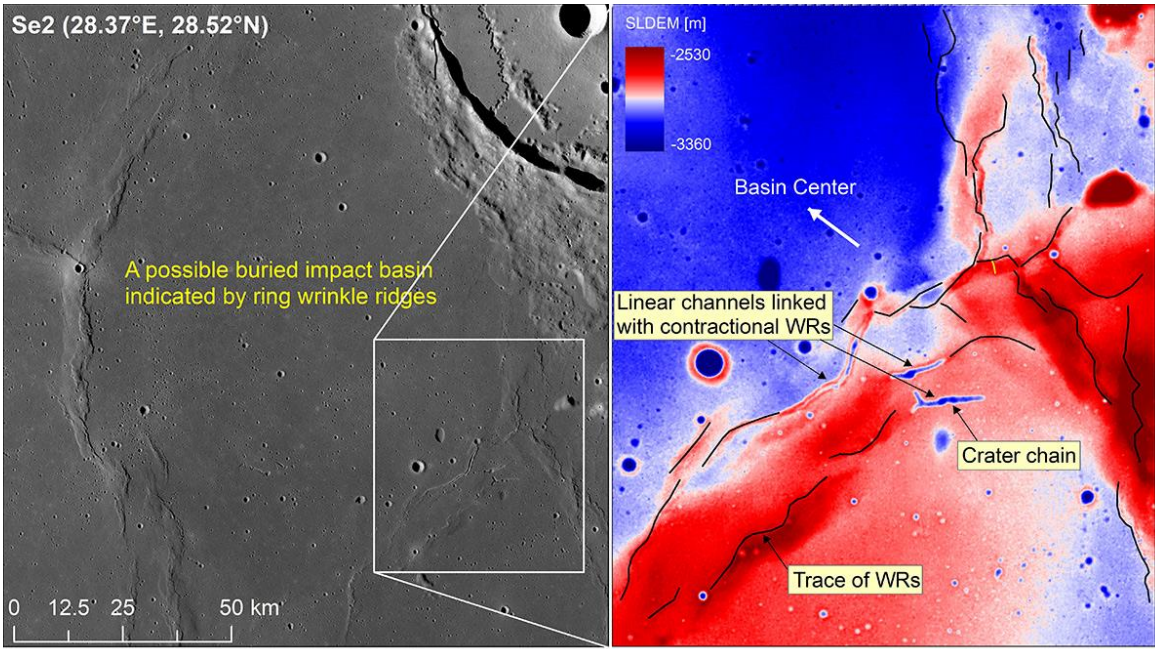

Supplementary Figure 13. (left) Kaguya TC map for the geologic background of the features investigated in the figure (right): the top-left channel is interpreted as a sinuous rille which is roofed at both its proximal and distal ends as suggested by Coombs and Hawke (23). Linear depression containing a chain of craters is always thought to be related to magmatic processes (e.g., near-surface gas-controlled venting process due to dike propagation) (24). The arrangement of these eruption centers and the strike of wrinkle ridges appear to be controlled by the subsurface normal faults/fractures concentric to the inferred basin center.

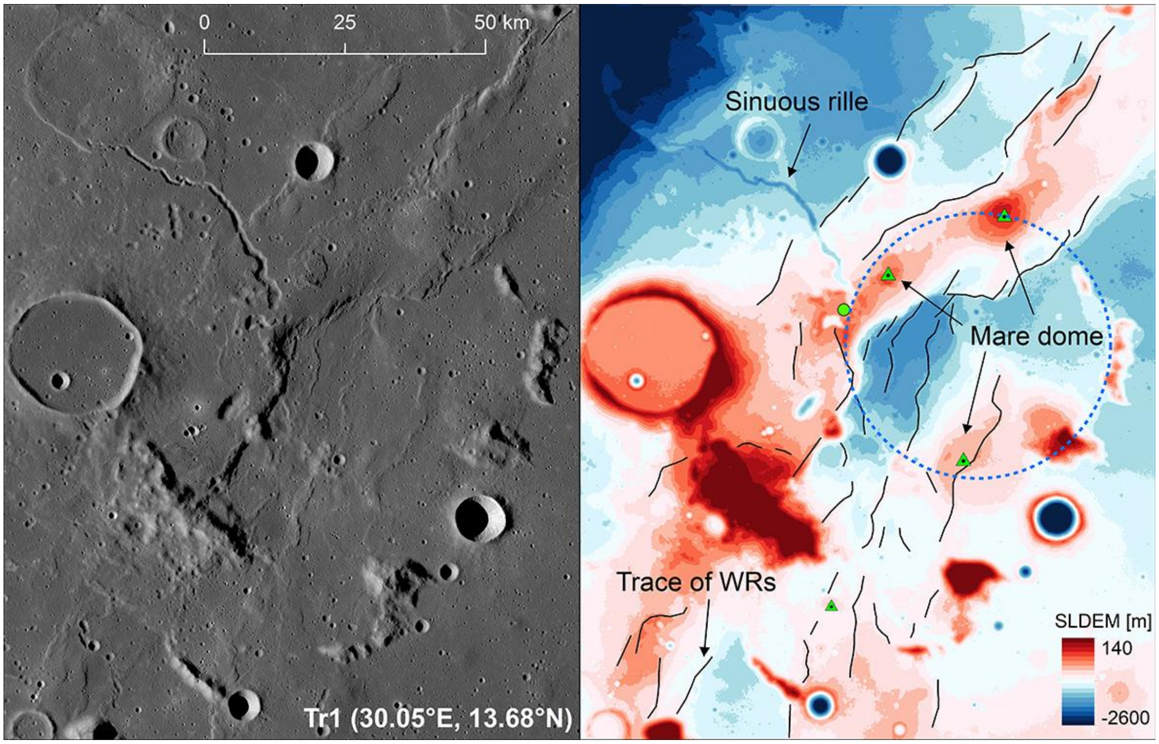

Supplementary Figure 14. These volcanic features are well situated on the broad arch of the major WR, which together with multiple elongated hills form a ring and was interpreted as a possible buried impact crater confined from gravity (Evans et al. (25); Zhang et al. (26) and their figures 2a and 3a). On the right topographic map, the arrangement of the three mare domes (green triangle), the rille head crater (green circle), and the three elongated hills to the right together forms a ring (blue dashed circle), suggesting a possible subsurface ring structural control of the WR-hosting eruptions.

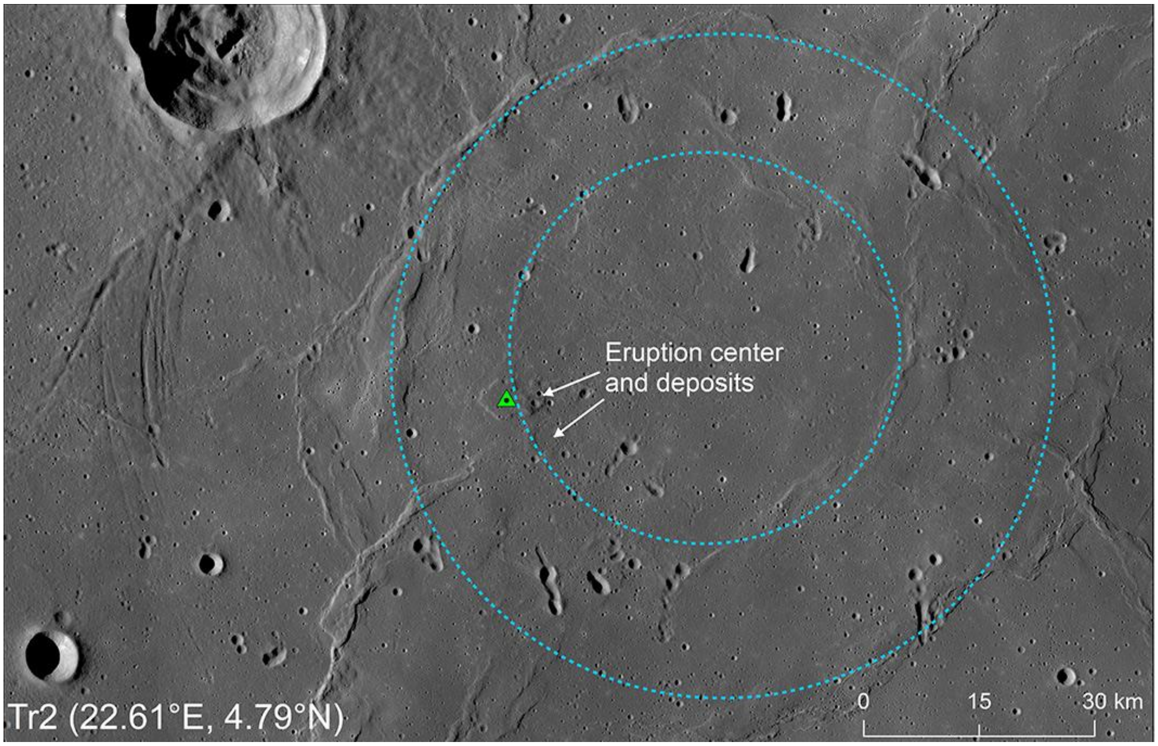

Supplementary Figure 15. The eruption center and lava deposits are located near the inner ridge ring (inner blue dashed circle) of the Lamont mascon (Neumann et al. (27)) in the western Mare Tranquillitatis. The geologic details of the volcanic features are available by Zhang et al. (26) and their figs. 15-17.

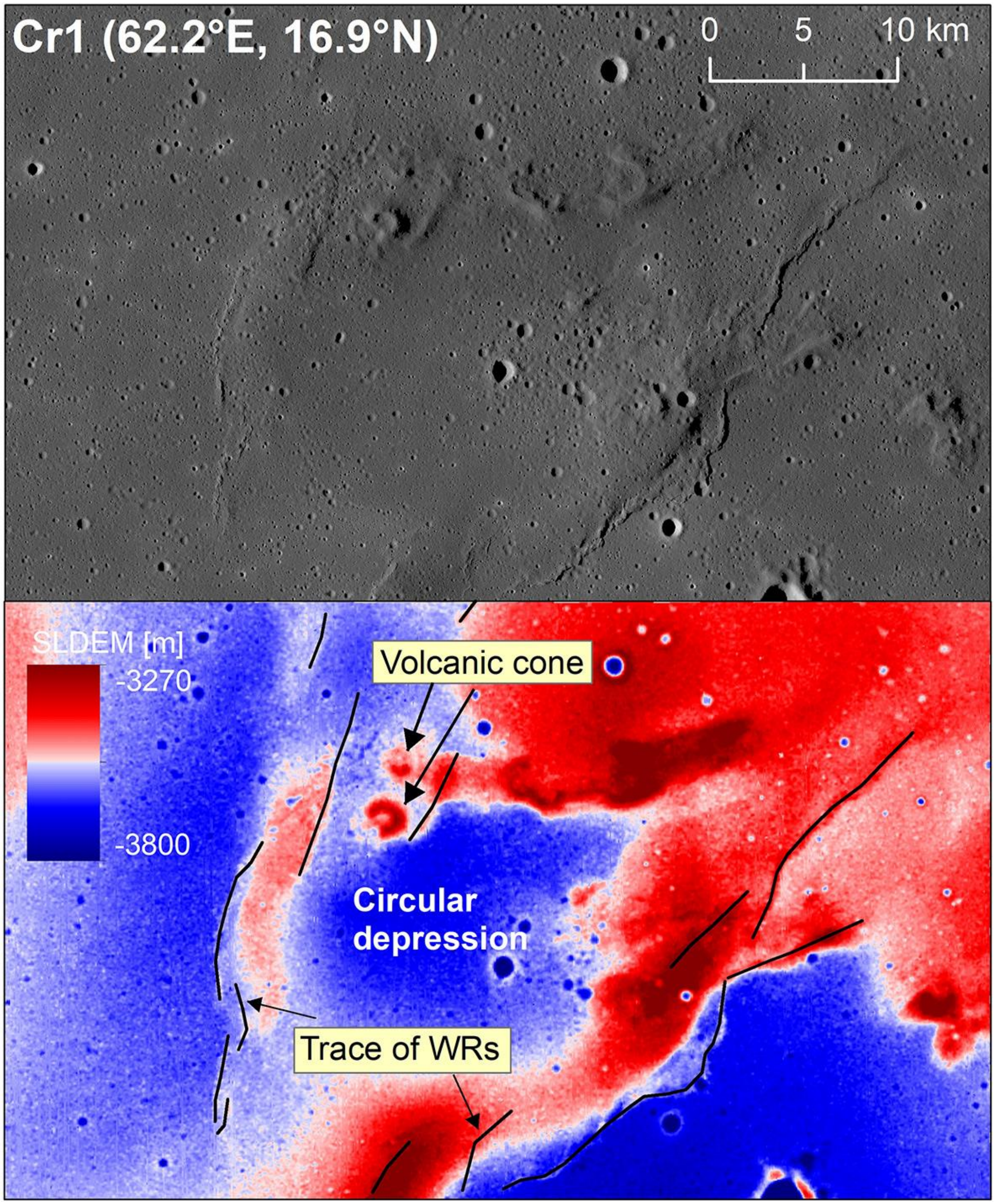

Supplementary Figure 16. The circular depression is characterized by a ring composed of WRs, and the type of this structure is always interpreted as a buried crater. The two volcanic cones are situated at the rim zone of the depression and also between two wrinkle ridge segments.

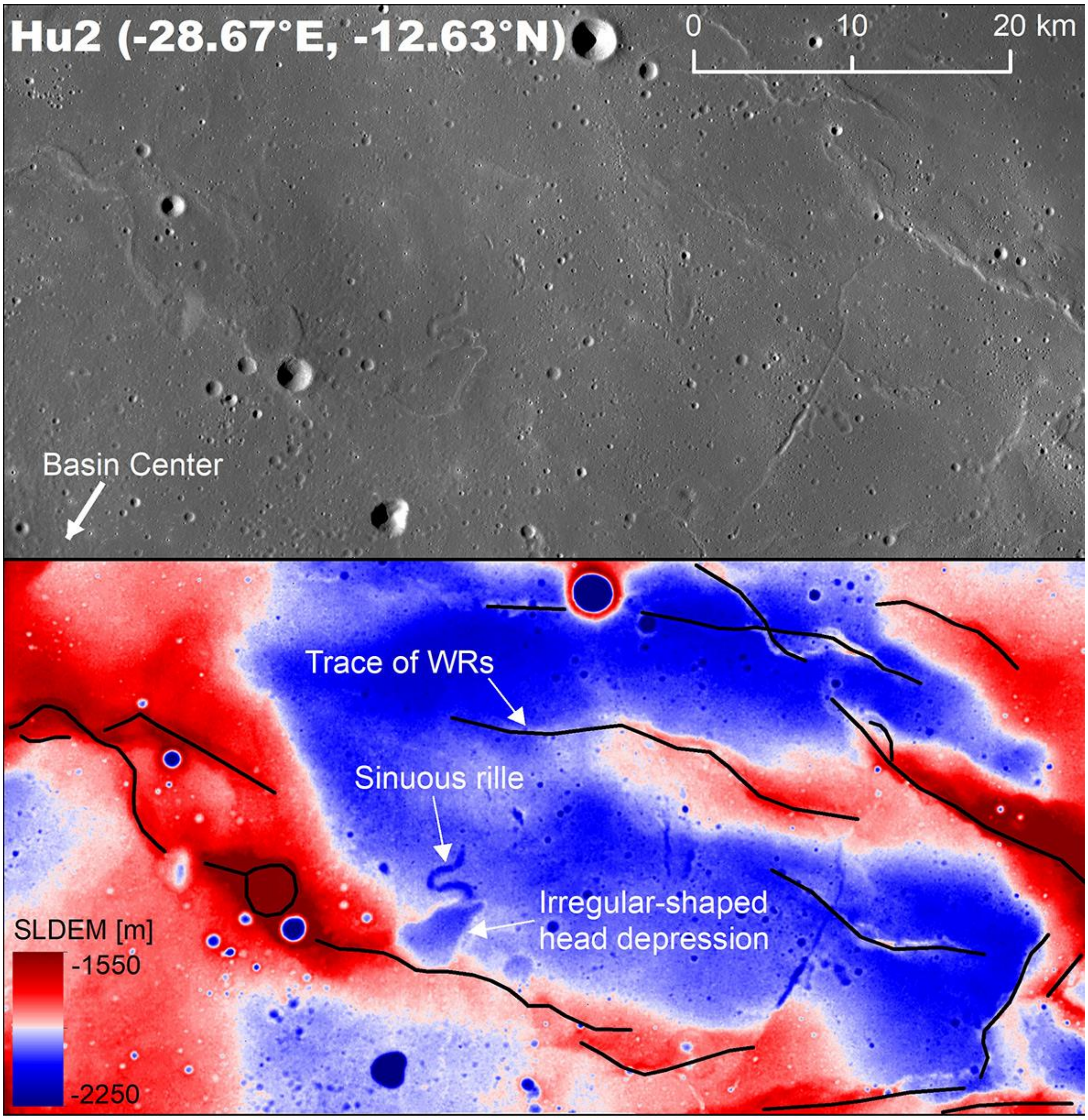

Supplementary Figure 17. The southwest rim of the irregular-shaped depression of the rille is along the strike of the ridge nearby. These WRs are concentric to the Humorum basin center.

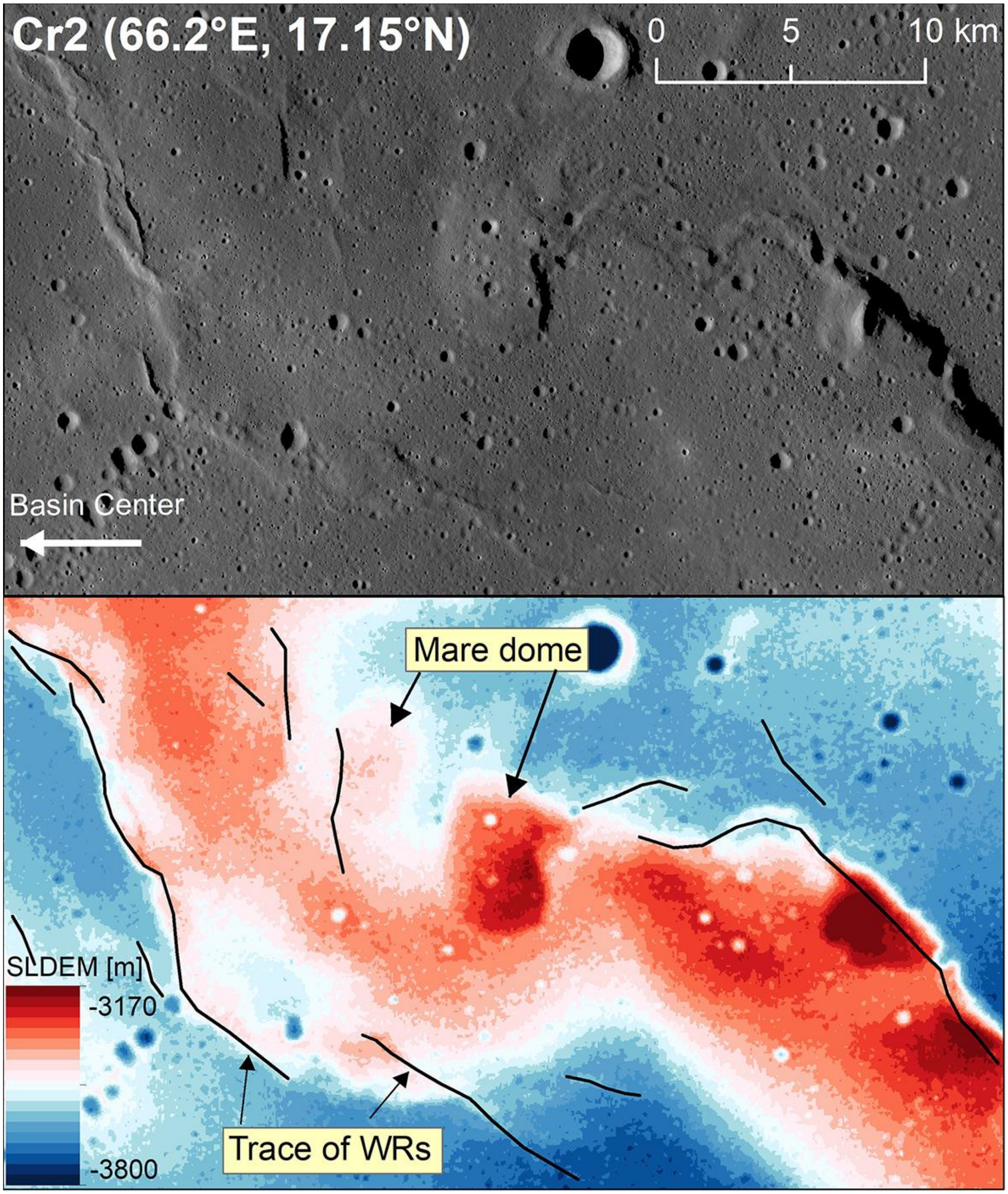

Supplementary Figure 18. The two mare domes are located on the broad arch of WRs, which are concentric to the Crisium basin center.

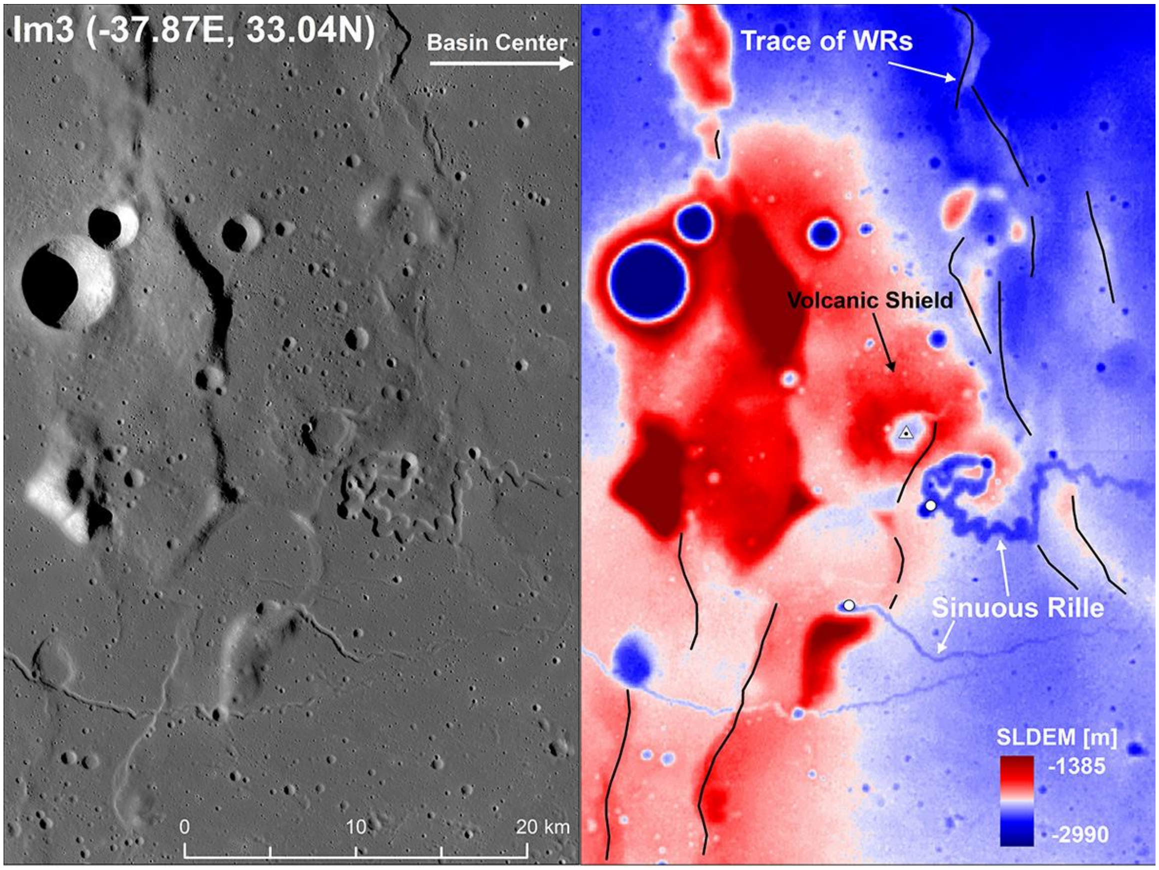

Supplementary Figure 19. A volcanic shield and two sinuous rilles with their head craters are located on the broad arch of WRs, which is concentric to the Imbrium basin center.

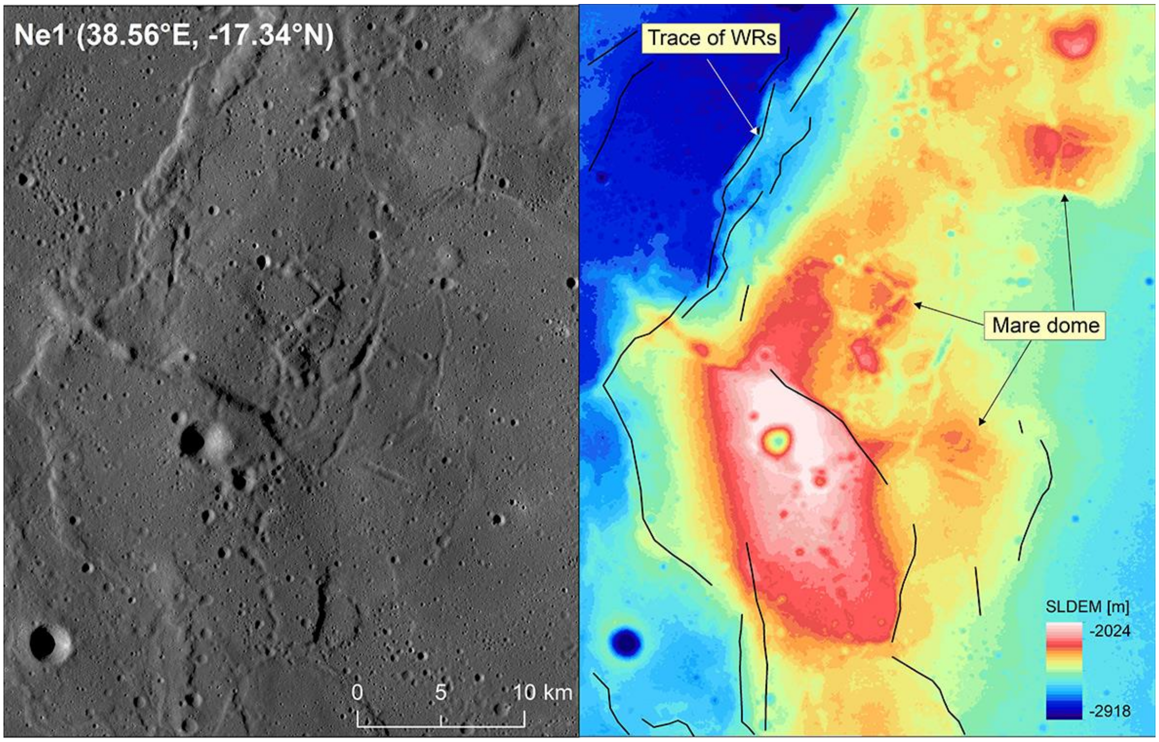

Supplementary Figure 20. These mare domes are located on the broad arch (rise) of WRs, which are concentric to the Nectaris basin center.

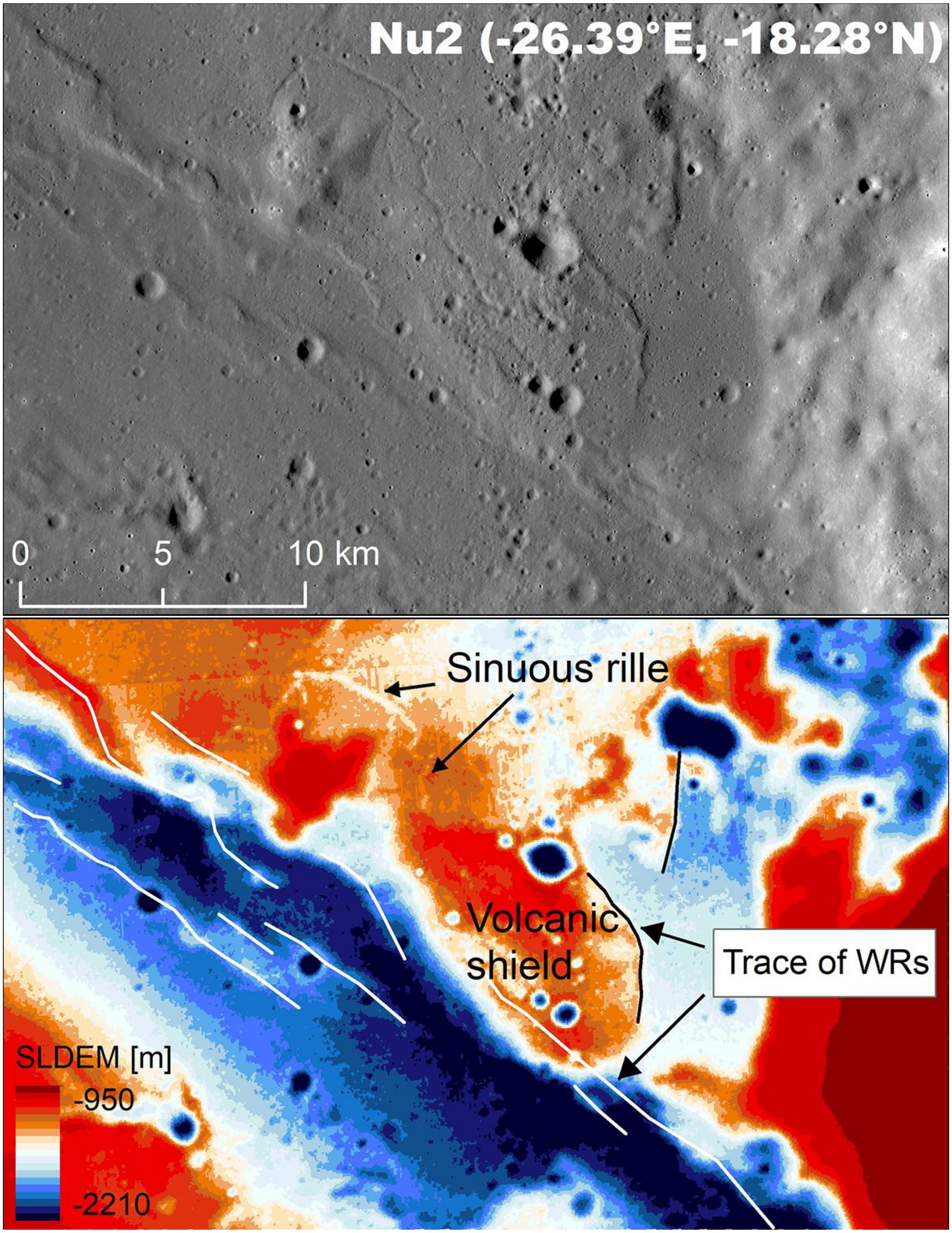

Supplementary Figure 21. A sinuous rille emanates from the volcanic rise (shield) and its boundary is clearly constrained by WRs, which are concentric to the Nubium basin center.

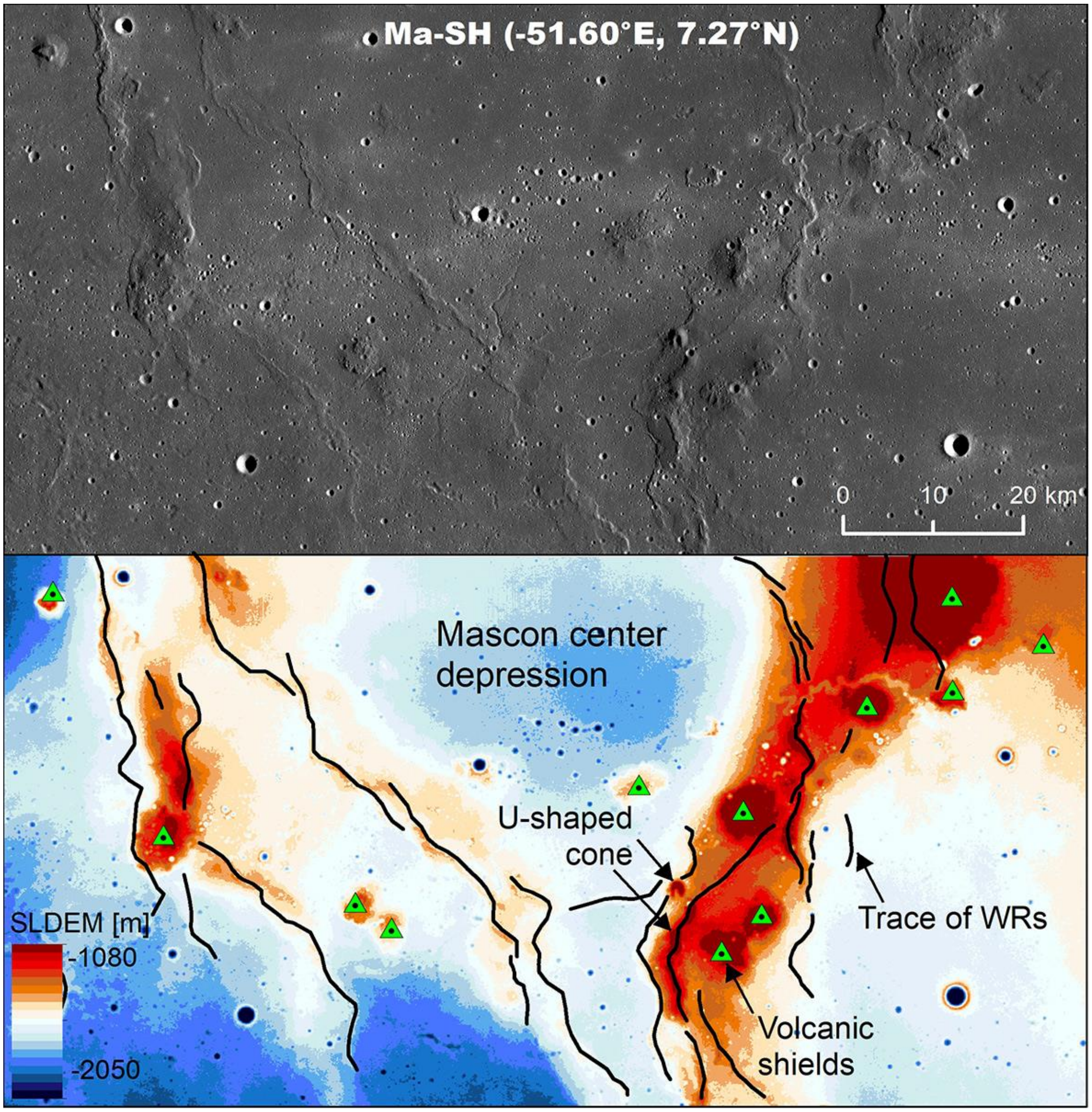

Supplementary Figure 22. Many volcanic shields are located on the broad arches of WRs, which form a major ring ridge above a buried impact crater rim, inferred and defined from gravity (see Evans et al. (25); Zhang et al. (26) and their figures 10 and 12).

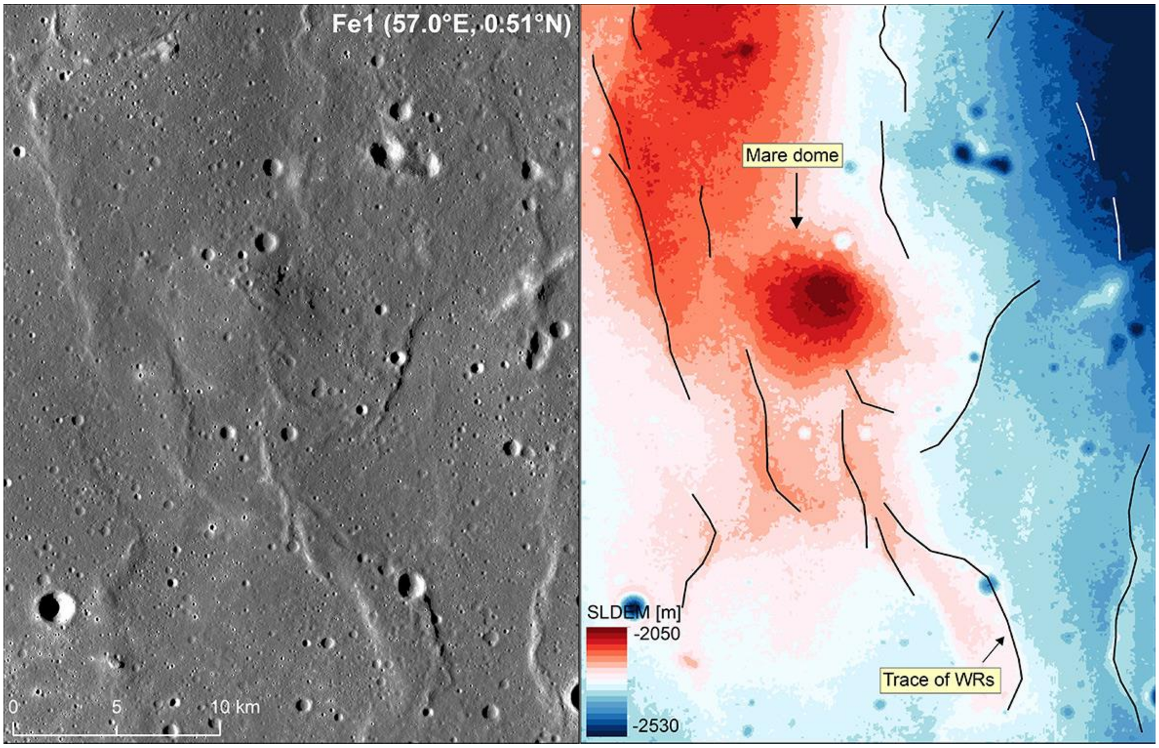

Supplementary Figure 23. The mare dome is located at a zone linked with several segments of WRs.

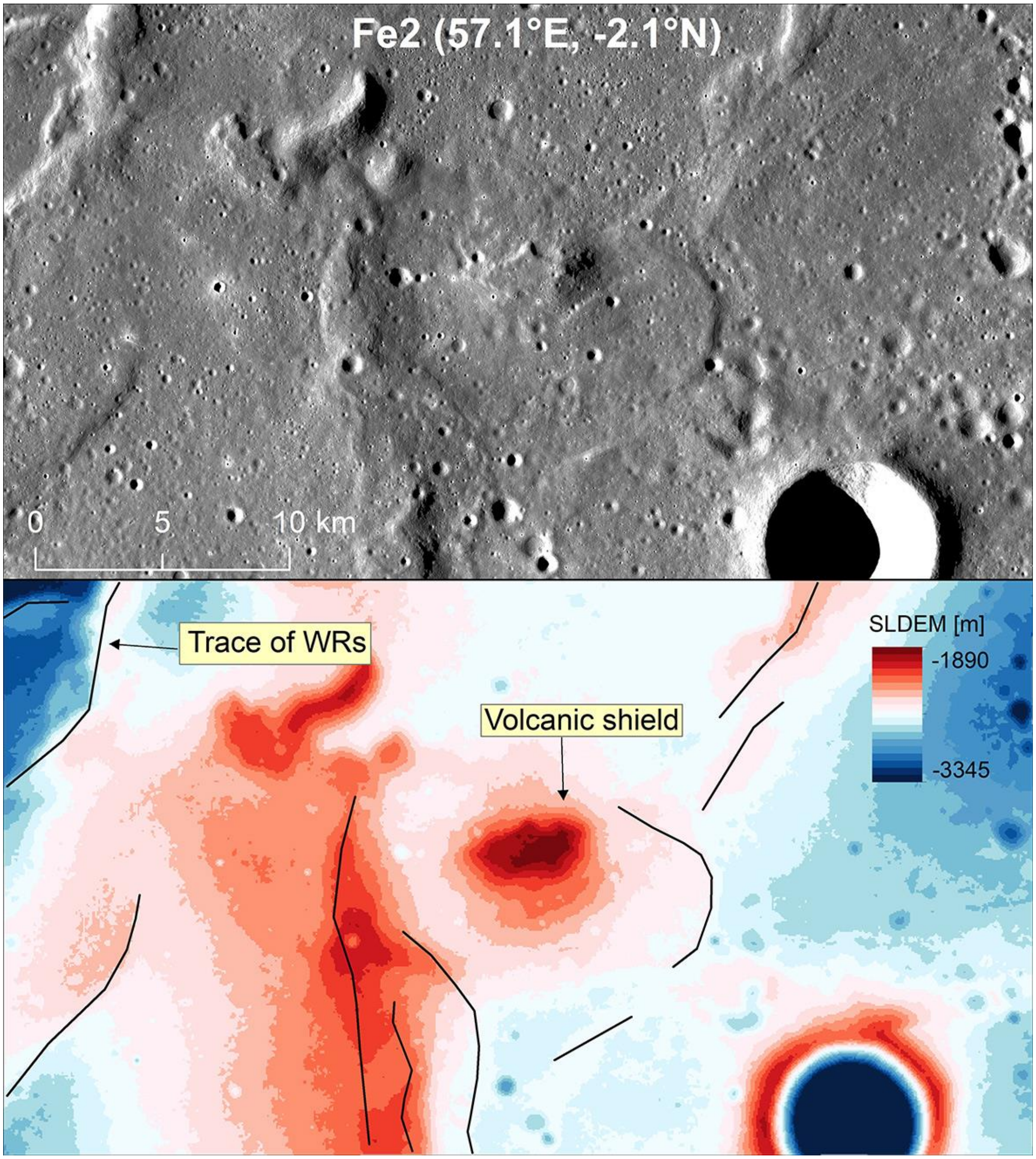

Supplementary Figure 24. The volcanic edifice is located in a region with complex pattern of WRs.

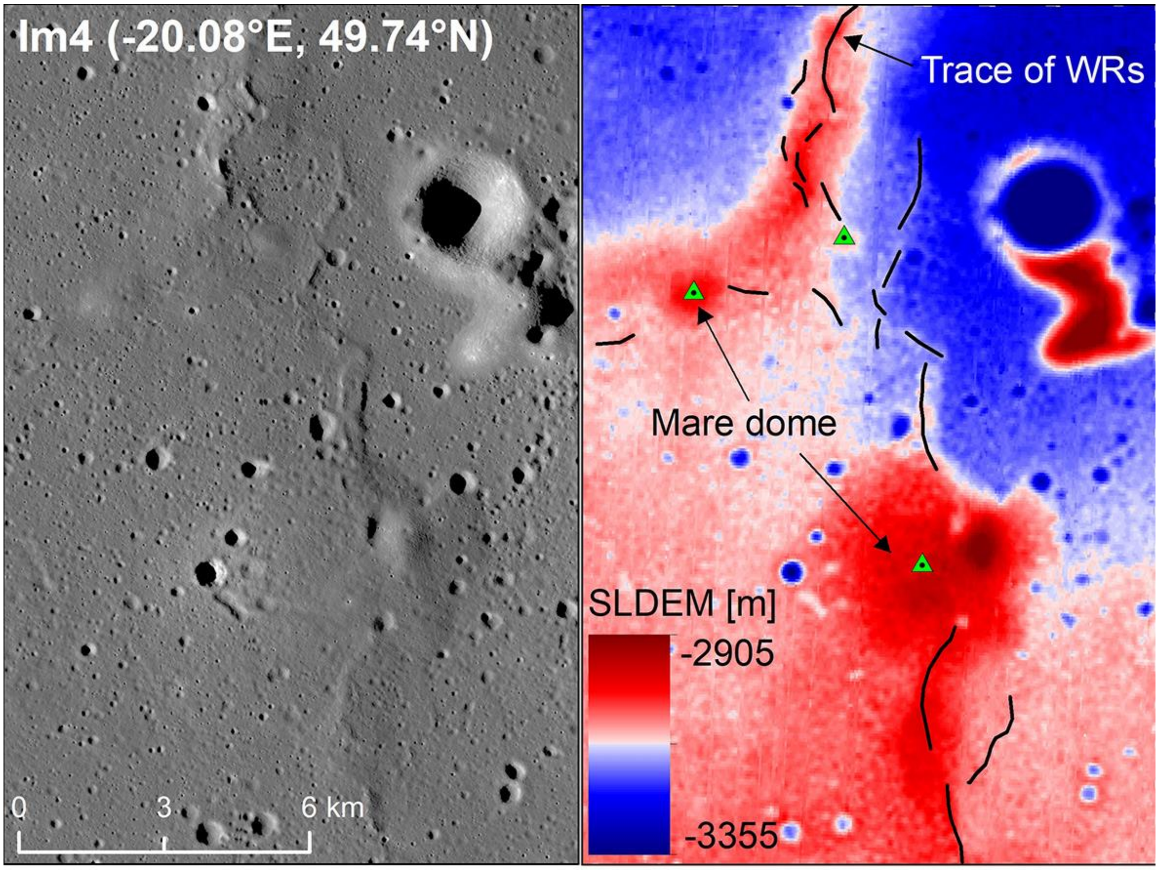

Supplementary Figure 25. Spatial relationship between three small shields and WRs**.**

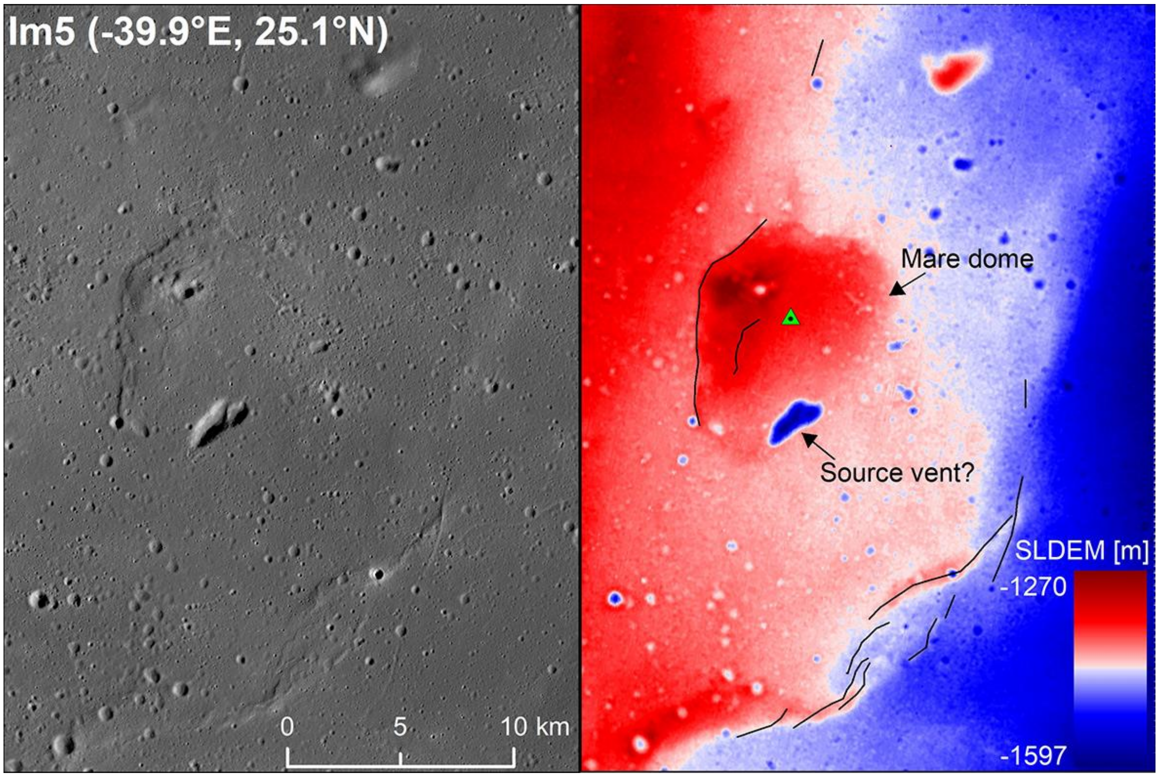

Supplementary Figure 26. The western border of the dome is well confined by the WR, suggesting a structural control of the dome-forming lava emplacement.

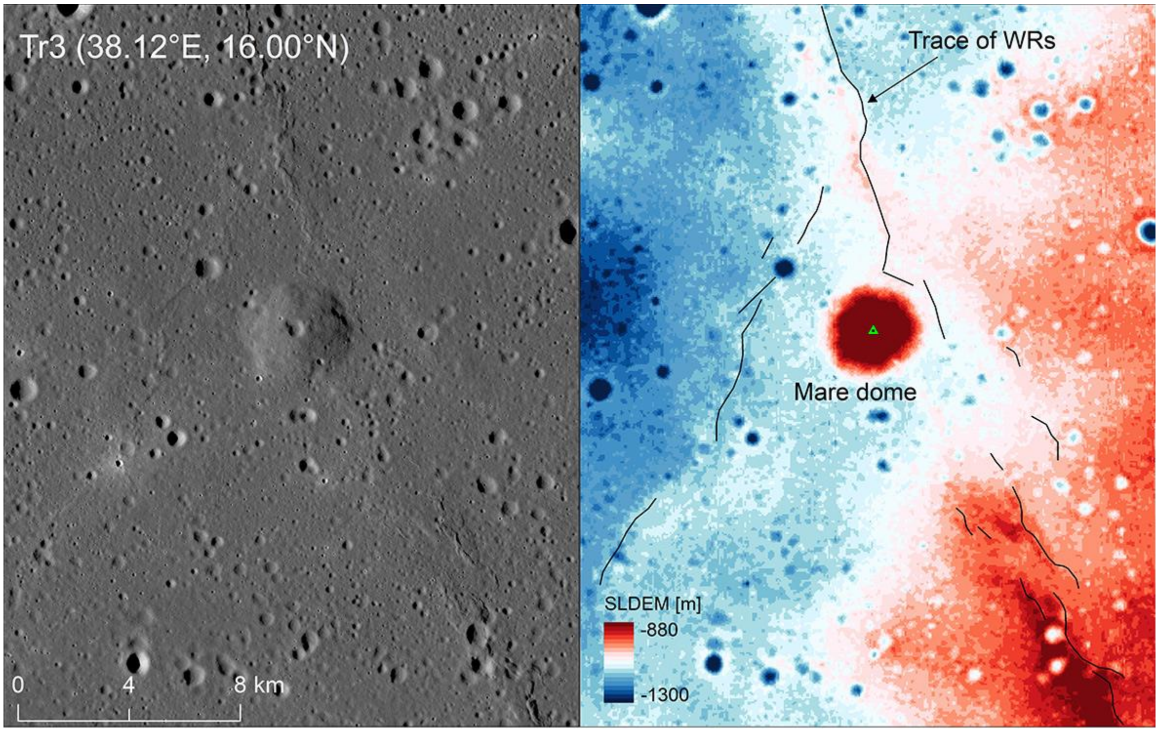

Supplementary Figure 27. The mare dome is located in a zone linked with WRs.

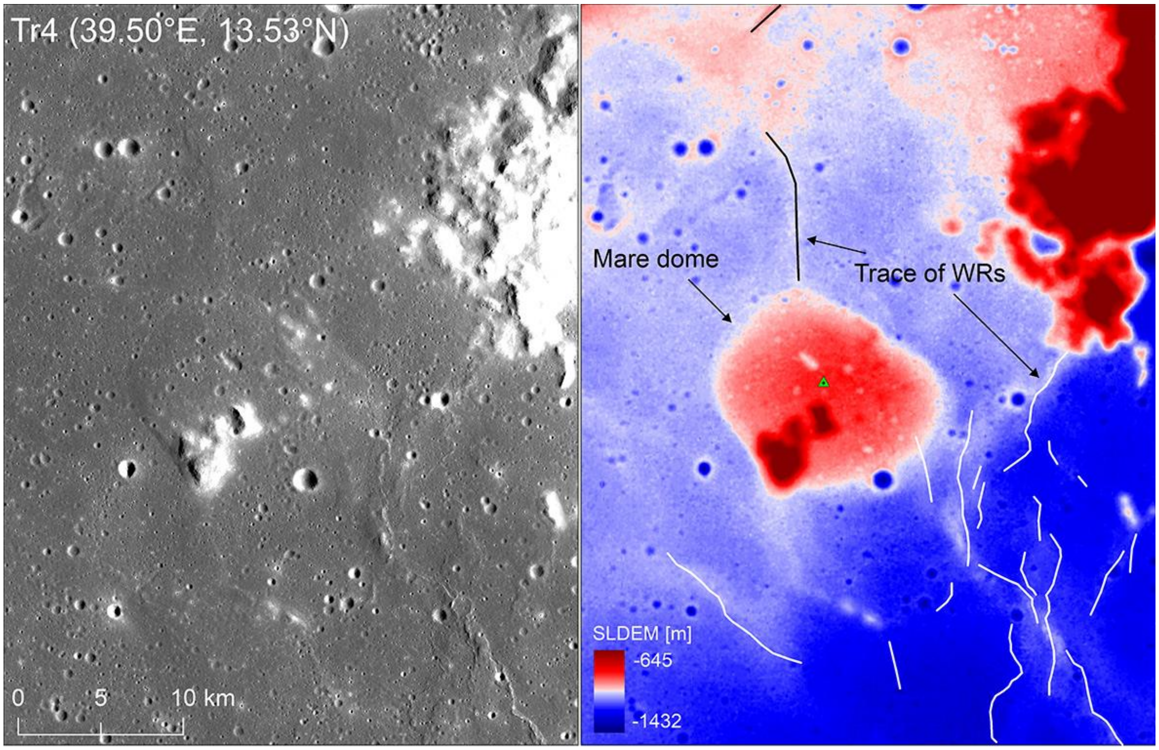

Supplementary Figure 28. The mare dome is located in a zone linked with WRs.

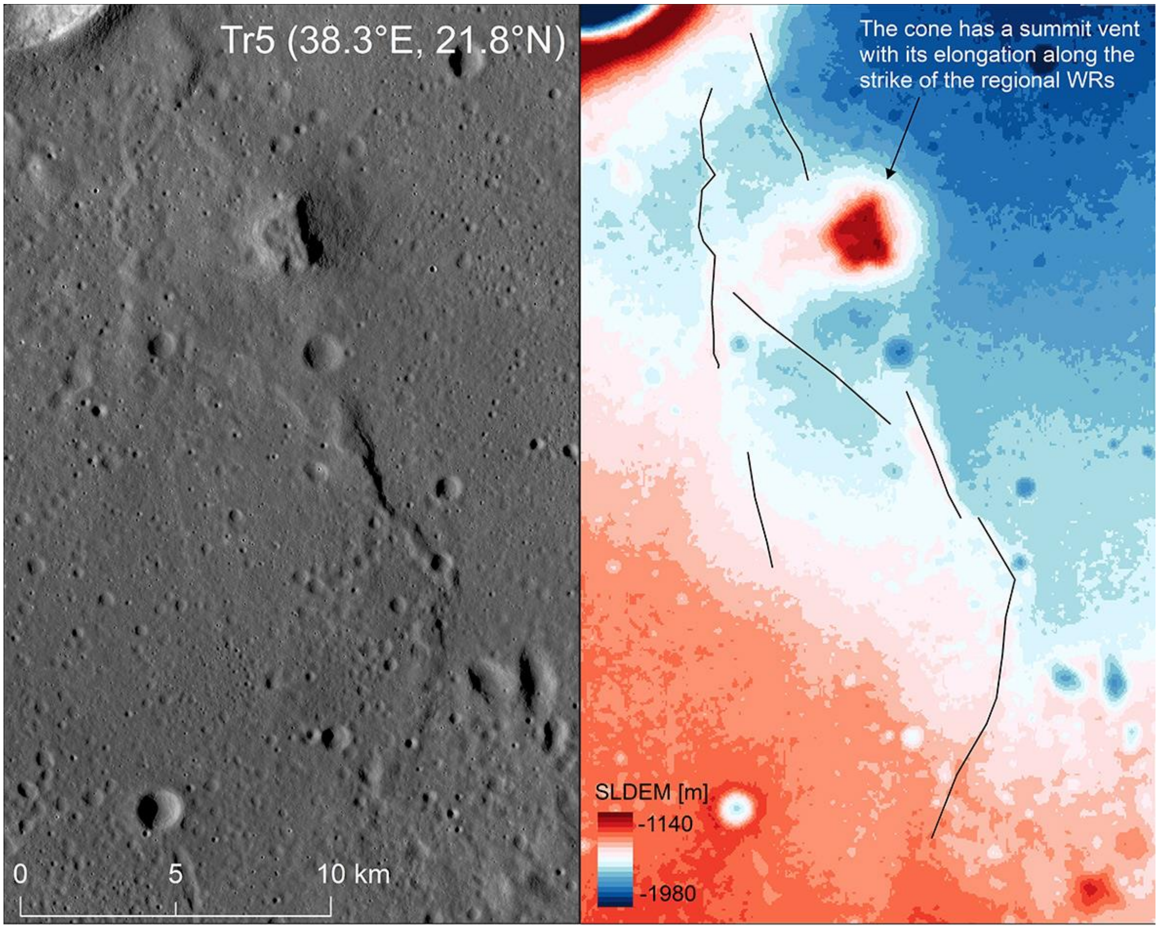

Supplementary Figure 29. The volcanic cone is located in a zone linked with WRs.

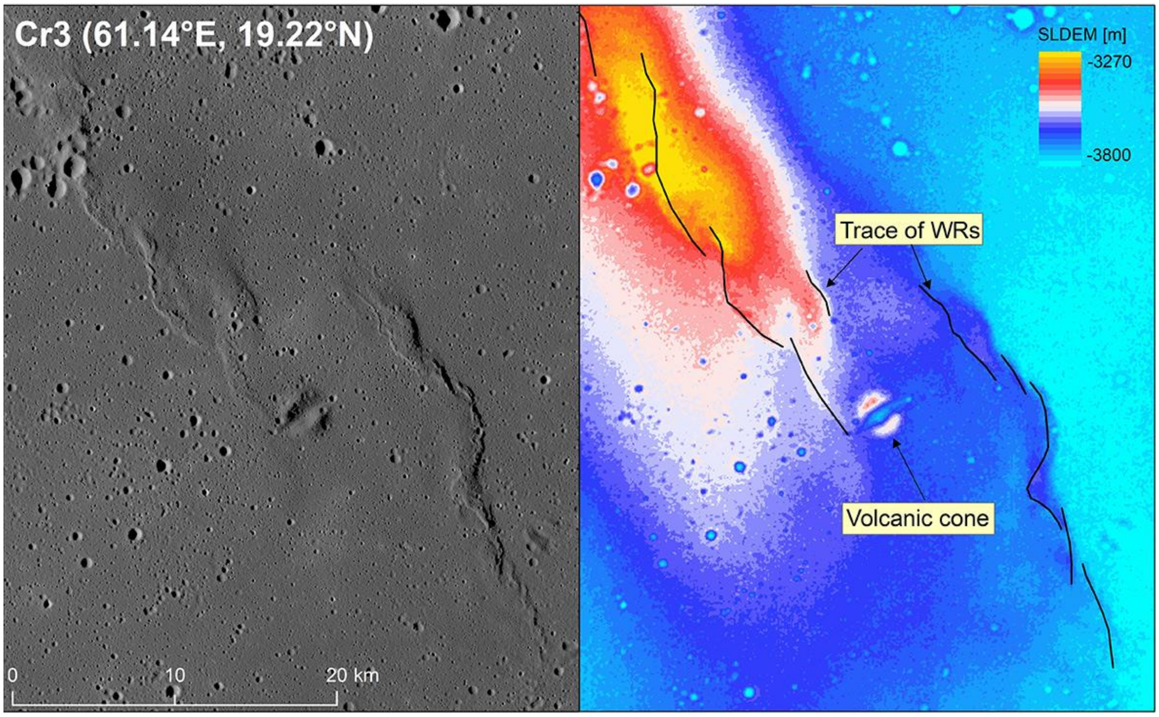

Supplementary Figure 30. Note that the elongation of the summit crater of the cone is along the transfer zone between *en* echelon-patterned WRs.

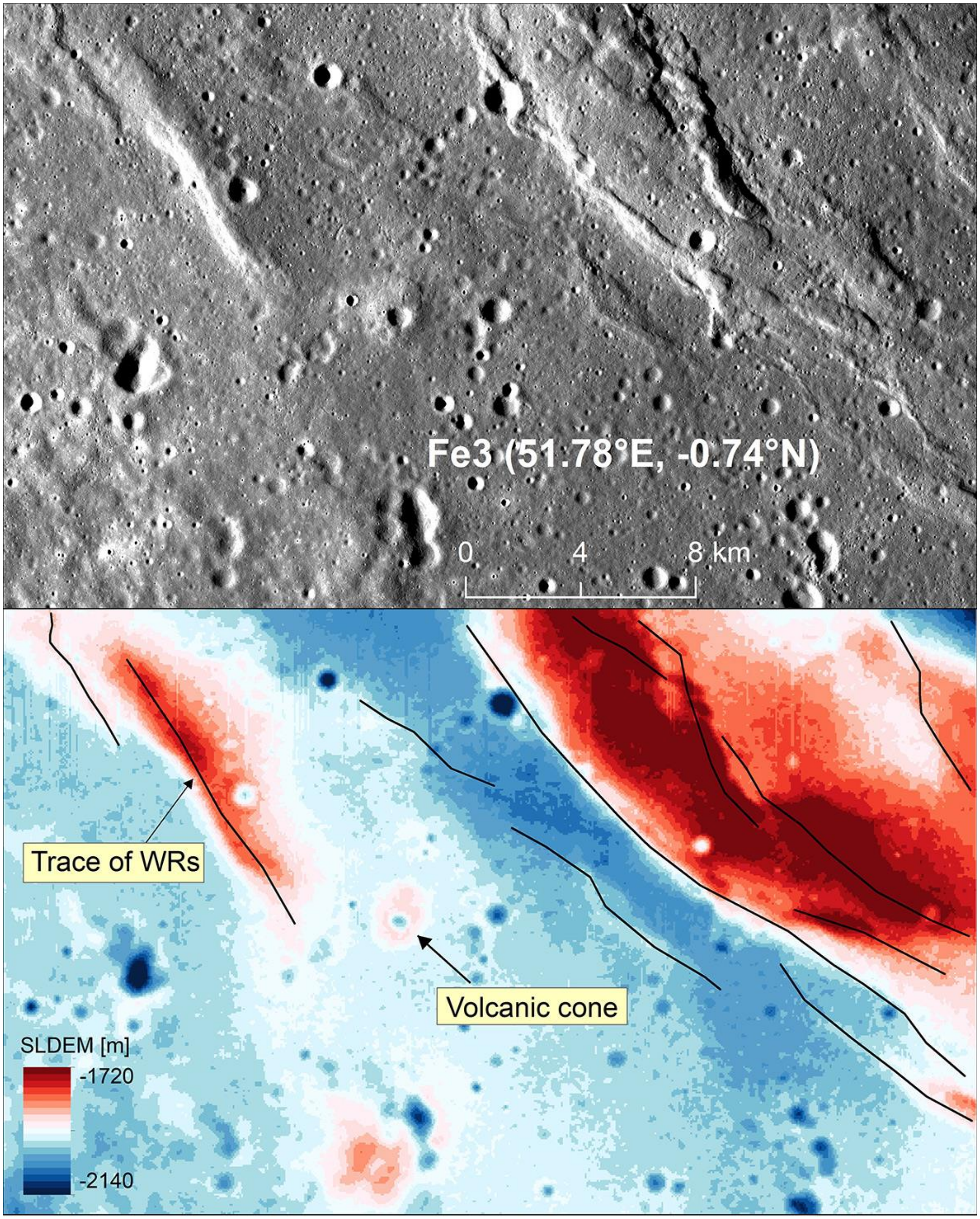

Supplementary Figure 31. The volcanic cone is located in a region linked with WRs.

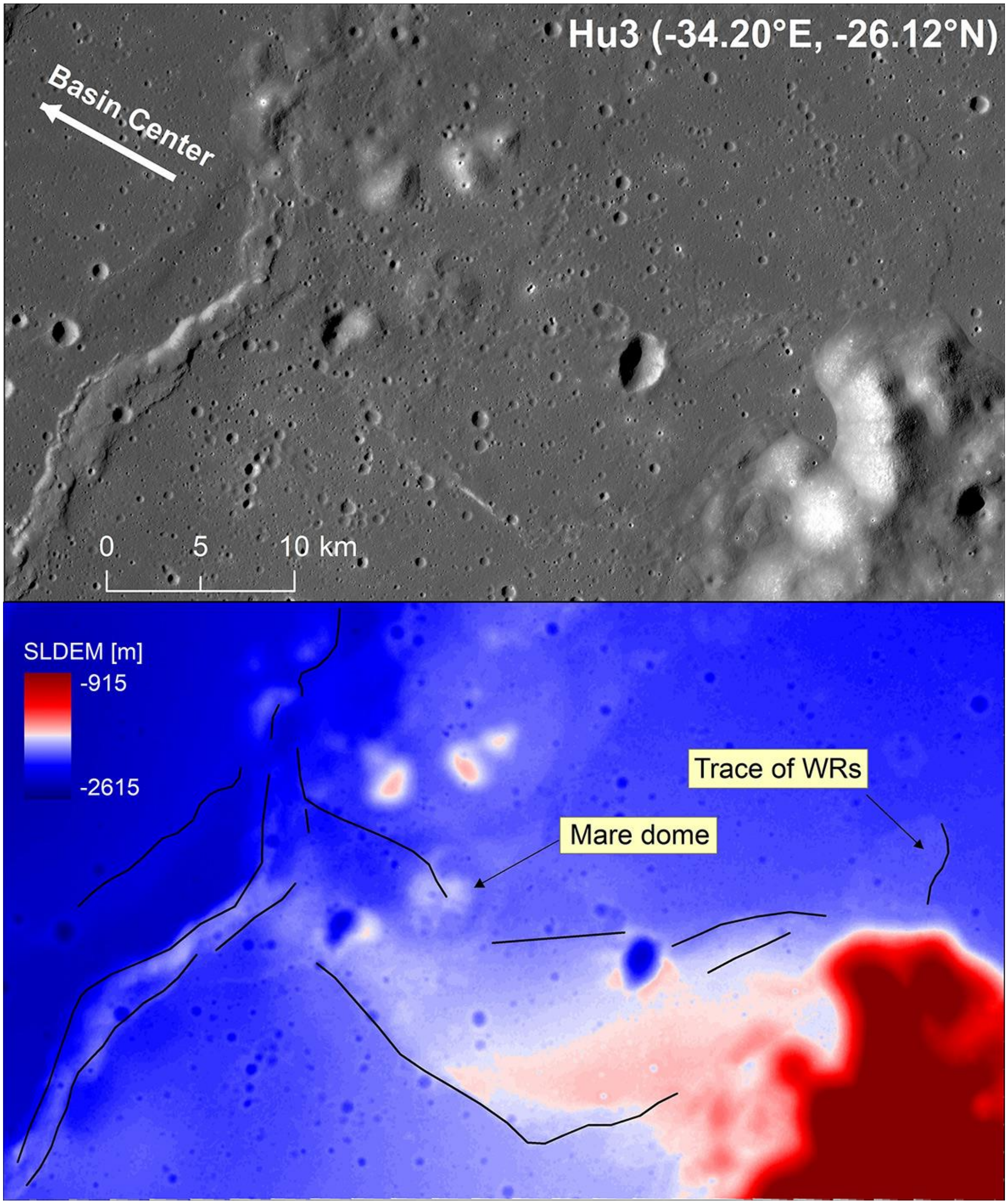

Supplementary Figure 32. The mare dome is at the zone linked with WRs.

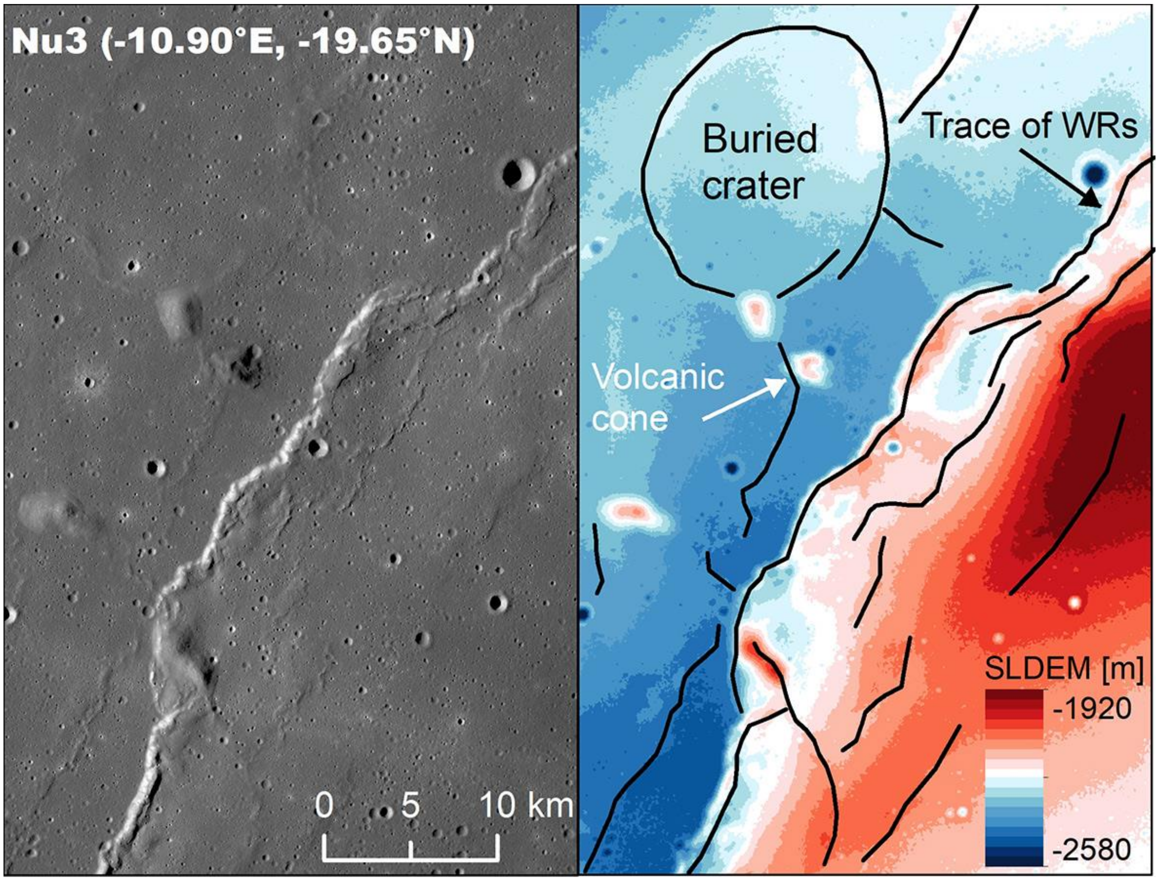

Supplementary Figure 33. The volcanic cone occurs in a complex WR-pattern zone.

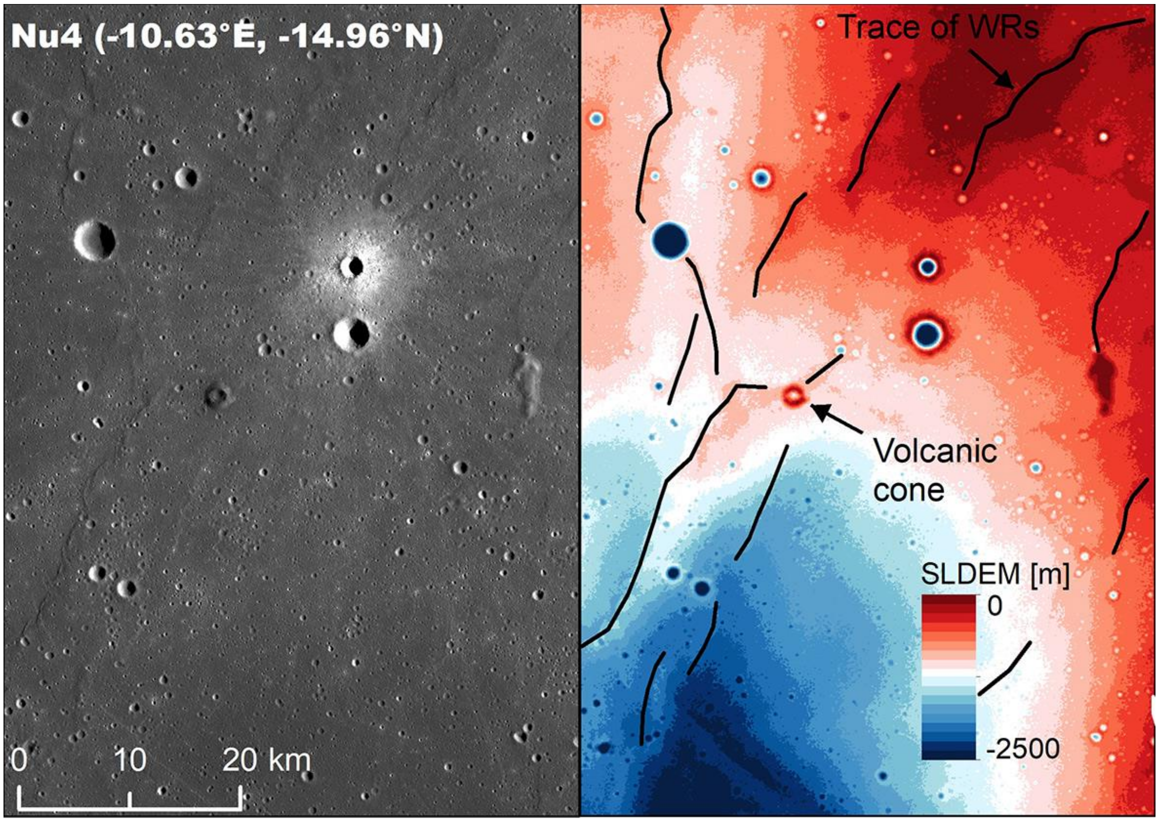

Supplementary Figure 34. The topographically well-preserved volcanic cone is located in a zone linked with WRs.

References

1. Wilhelms, D. E. The Geologic History of the Moon. *US Geol. Survey Prof. Pap*. 1348 (1987).
2. Neukum G., Ivanov B.A., & Hartmann W. K. Cratering Records in the Inner Solar System in Relation to the Lunar Reference System. In *Chronology and Evolution of Mars* (eds Kallenbach R., Geiss J., & Hartmann W. K.) 12. (Springer, Dordrecht, 2001).
3. Stöffler, D. et al. Cratering history and lunar chronology. *Reviews in Mineralogy and Geochemistry*, 60(1), 519-596 (2006).
4. Wilhelms, D. E., & McCauley, J. F. Geologic map of the near side of the Moon: U.S. Geological Survey Miscellaneous Geologic Investigations Map I-703, scale l:5,000,000 (1971).
5. Fortezzo, C. M., & Hare, T. M. Completed digital renovation of the 1: 5,000,000 lunar geologic map series. In *44th Lunar Planet. Sci. Conf.* abstr. 2114 (2013).
6. Wilhelms, D. E., & El-Baz, F. Geological Map of the East Side of the Moon: U.S. Geological Survey Miscellaneous Geologic Investigations Map I-948, scale l:5,000,000 (1977).
7. Scott, D. H., & McCauley, J. F. Geologic map of the west side of the Moon: U.S. Geological

Survey Miscellaneous Geologic Investigations Map I-1034, scale l:5,000,000 (1977).

8. Lucchitta, B.K. Geologic map of the north side of the Moon: U.S. Geological Survey Miscellaneous Geologic Investigations Map I-1062, scale l:5,000,000 (1978).
9. Wilhelms, D. E., Howard, K. A., & Wilshire, H. G. Geologic map of the south side of the Moon: U.S. Geological Survey Miscellaneous Geologic Investigations Map I-1162, scale l:5,000,000 (1979).
10. Greeley, R. & Batson, R. The NASA Atlas of the Solar System, Cambridge Univ. Press, Cambridge, 369 (1997).
11. Tanaka, K. L., Skinner, J. A., & Hare, T. M. Planetary Geologic Mapping Handbook-2010: U.S. Geological Survey, Astrogeology Science Center 2255 N. Gemini Dr., Flagstaff, AZ 86001 (2010).
12. Gaddis, L., Tanaka, K., Skinner, J. & Hawke, B. R. Lunar geologic mapping program: 2008 update. In Annu. Meeting Planet. Geol. Mappers, Document ID: 20080041022 (2008).
13. Robinson, M. S. et al. Lunar reconnaissance orbiter camera (LROC) instrument overview. *Space Sci. Rev.* **150**, 81-124 (2010).
14. Scholten, F. et al. GLD100: The near-global lunar 100 m raster DTM from LROC WAC stereo image data. *J. Geophys. Res. Planets* **117**, E00H17 (2012).
15. Sato, H. et al. Lunar mare TiO_2 abundances estimated from UV/Vis reflectance. *Icarus* **296**, 216-238 (2017).
16. Zuber, M. T. et al. Gravity field of the Moon from the Gravity Recovery and Interior Laboratory (GRAIL) mission. *Science* **339**(6120), 668-671 (2013).
17. Barker, M. K. et al. A new lunar digital elevation model from the Lunar Orbiter Laser Altimeter and SELENE Terrain Camera. *Icarus* **273**, 346–355 (2016).
18. Haruyama, J. et al. Planned radiometrically calibrated and geometrically corrected products of lunar high-resolution Terrain Camera on SELENE. *Adv. Space Res.***42**(2), 310-316 (2008a).
19. Haruyama, J. et al. Global lunar-surface mapping experiment using the Lunar Imager/Spectrometer on SELENE. *Earth Planet. Space*, **60**(4), 243-255 (2008b).
20. Thompson, T.J. et al. Global lunar wrinkle ridge identification and analysis. In *48th Lunar Planet. Sci. Conf.* abstr. 2665 (2017).
21. Wieczorek, M. A. et al. The crust of the Moon as seen by GRAIL. *Science* **339**(6120), 671-675 (2013).
22. Goossens, S. et al. High-Resolution Gravity Field Models from GRAIL Data and Implications for Models of the Density Structure of the Moon's Crust. *J. Geophys. Res. Planets* **125**, e2019JE006086 (2020).

23. Coombs, C. R. & Hawke, B. R. A search for intact lava tubes on the Moon: Possible lunar base habitats. In *Lunar Planet. Sci. Conf.*, 219-229 (1992).
24. Head, J. W., & Wilson, L. Generation, ascent and eruption of magma on the Moon: New insights into source depths, magma supply, intrusions and effusive/explosive eruptions (Part 2: Predicted emplacement processes and observations). *Icarus* **283**, 176-223 (2017).
25. Evans, A. J. et al. Identification of buried lunar impact craters from GRAIL data and implications for the nearside maria. *Geophys. Res. Lett.* **43**(6), 2445-2455 (2016).
26. Zhang, F. et al. Diversity of basaltic lunar volcanism associated with buried impact structures: Implications for intrusive and extrusive events. *Icarus* **307**, 216-234 (2018).
27. Neumann, G. et al. Lunar impact basins revealed by Gravity Recovery and Interior Laboratory measurements. *Sci. Adv.* **1** (9), e1500852 (2015).
